# Supplementary material for: Equipping community pharmacy workers as agents for health behaviour change: developing and testing a theory-based smoking cessation intervention
Source: BMJ Open. 2017 Aug 11;7(8):e015637. doi: 10.1136/bmjopen-2016-015637 (PMC5724215; doi:10.1136/bmjopen-2016-015637)
Supplement: Supplementary file 1 [file bmjopen-2016-015637supp001.pdf]

Supplementary Table 1. Summary of preliminary findings from realist review

Mechanism by which the pharmacy smoking cessation service might be promoted	How the mechanism might be strengthened	Contextual influences
**Pharmacist identity**	• Strengthen 'pharmacy' identity by emphasising backing from professional bodies<br>• Promote non-medication and public health roles of the pharmacist<br>• Encourage patient-orientation rather than product-orientation<br>• Encourage a professional as opposed to 'technical' ethos	• Undergraduate education promotes these characteristics<br>• Professional bodies embrace extended role<br>• Policymakers recognise pharmacists as professionals
**Pharmacist capability**	• Strengthen knowledge base on health behavior change<br>• Consultation skills training<br>• Easily accessible educational sessions<br>• Change beliefs and attitudes, boosting self-efficacy in delivering the smoking cessation and encouraging belief that the intervention will be effective	• Quality, depth and breadth of training<br>• Training addresses skills and attitudes as well as knowledge<br>• Accessibility of training throughout professional life
**Pharmacist motivation**	• Present business arguments eg diversification of revenue streams, investment in space for financial returns<br>• Recognise training as continuing professional development	• Involvement of other pharmacies and pharmacists in health behavior change establishing a professional norm.<br>• Strong business model justifying investment in infrastructure<br>• Simple system for claiming payments
**Stakeholder confidence**	• Build confidence in the intervention from government, professional bodies (general practitioner and pharmacy), health commissioners.<br>• Change perceptions of patients and carers about the position of the pharmacist in health care system.	• Clear, positive messages in the media about the extended role<br>• Positive reaction to the role from other branches of primary care at national and local level
