# Supplementary material for: Equipping community pharmacy workers as agents for health behaviour change: developing and testing a theory-based smoking cessation intervention
Source: BMJ Open. 2017 Aug 11;7(8):e015637. doi: 10.1136/bmjopen-2016-015637 (PMC5724215; doi:10.1136/bmjopen-2016-015637)
Supplement: Supplementary file 2 [file bmjopen-2016-015637supp002.pdf]

Supplementary Table 2. The intervention to be evaluated in the STOP randomised controlled trial described using the Template for Intervention Description and Replication (TIDieR) checklist.*

**Item 1. Brief name: Provide the name or a phrase that describes the intervention**	STOP - Service optimisation and communication skills training to strengthen the NHS smoking cessation service in community pharmacies.
**Item 2. Why: Describe any rationale, theory, or goal of the elements essential to the intervention**	We used the COM-B as a starting framework recognising the three behaviours of i) engaging smokers, ii) retaining smokers in the service and iii) smokers quit rates. For each we applied further theories including Social Cognitive theory [26] to underpin development of the intervention. The intervention aims to boost self-efficacy and self-regulation in the pharmacy staff to improve skills promoting recruitment and retention of service users and increasing quit success. We also drew upon Self Determination theory[27] to influence motivation which we see as important for the pharmacy staff to react to opportunities to promote the service and to create opportunities to discuss the service with potential clients.<br><br>We mapped the theoretical constructs onto specific behaviour change techniques using the behaviour change wheel and COM-B behaviour change model.[19 20] This model is increasingly used in development of behavioural interventions and has previously been used for smoking cessation. These behaviour change techniques were then embedded within the intervention.<br><br>*For implementation of the intervention we applied Diffusion of Innovations theory [34] which enabled us to seat the intervention in the complex organisational structures which we found both within and between community pharmacies.*<br><br>*The training stresses the advantages over usual practice in terms of revenue for the pharmacy and in building up the common good. The intervention is brief and fits in with usual practice and work flows and can be adapted in the context of each individual pharmacy. Concerns will be addressed about risks to profits – more important for the pharmacist/owners than for the staff. Backing from local opinion leaders will be emphasised (Hemant Patel, Secretary of the Local Pharmaceutical Committee) and we suggest that individual champions are appointed within each pharmacy perhaps also with a monthly 'STOP smoking day' to*

	maintain the focus of attention.
**Item 3. What (materials): Describe any physical or informational materials used in the intervention, including those provided to participants or used in intervention delivery or in training of intervention providers. Provide information on where the materials can be accessed**	Training material will be given as printed handouts in a study folder. The training sessions will involve role-plays and training videos – targeting engagement of smokers and optimising delivery of the stop smoking service aiming to increase retention and successful quitting. Attendees will be given a STOP flip chart (for desk) – with notes on key behaviour change techniques, 'Quit card' (can be kept in wallet) – to be given to smoker clients to reinforce smoking cessation strategies.<br><br>*The attendees will be given badges to wear in pharmacy as visual prompts to potential service users to ask about smoking cessation.*<br><br>*Social media will be used to perform a 'helpdesk' function using email and 'Whatsapp' to provide an easily accessible 'one-stop' platform to access study materials such as training videos. We will also signpost external smoking cessation resources using Whatsapp for example smoking cessation training websites, and latest cessation guidance.*<br><br>*All materials will be made available on a website to facilitate access by those not using social media.*
**Item 4. What (procedures): Describe each of the procedures, activities, and/or processes used in the intervention, including any enabling or support activities**	The training sessions will be face to face in a group of approximately 12 stop smoking advisors or pharmacy support staff. The STOP training will be incentivised for pharmacy staff by providing a cash amount of £30 to attend each session including travel expensed (£60 for both sessions) and a 'certificate of completion' to each staff member for attending and completing the training (see Appendix 2 and 3). The payment will be made only on completing the second session.
**Item 5. Who provided: For each category of intervention provider (for example, psychologist, nursing assistant), describe their expertise, background and any specific training given**	Training sessions will be facilitated by an experienced trainer/health psychologist and a community pharmacist, also a trainer. Skilled facilitation is needed for these groups, supported by the leader/co-leader structure and the pharmacists are then also available to answer practical questions about smoking cessation which we found arose frequently in the sessions that we conducted for the pilot study.
**Item 6. How: Describe the modes of delivery (such as face to face or by some other mechanism, such as**	The face-to-face training will be delivered at varying times to accommodate the work routine of pharmacy staff.<br><br>See also Item 3 above for internet and social media resources.

**internet or telephone) of the intervention and whether it was provided individually or in a group**	
**Item 7. Where: Describe the type(s) of location(s) where the intervention occurred, including any necessary infrastructure or relevant features**	The training will be delivered in training venues commonly used by pharmacy staff. Example of venues include the Tomlinson Centre in City and Hackney, Newham hospital or restaurants with separate meeting room in Newham. Venues will be chosen to be easily accessible to pharmacy workers after the pharmacy closes in the evening. Suggestions for venues will be taken from local Public Health commissioners and from the pharmacists themselves at recruitment.<br><br>Training sessions for pharmacy workers for other purposes are often held at restaurants where a meal or vouchers are provided and we will fit in with this model if possible if an appropriate meeting room is also available.
**Item 8. When and how much: Describe the number of times the intervention was delivered and over what period of time including the number of sessions, their schedule, and their duration, intensity or dose**	The training will comprise two sessions, each session of 2.5 hours, separated by two weeks. Pilot work suggested that longer sessions would not be more effective.<br><br>Tasks will be given at the end of the first session for advisors to complete NCSCT training (level 1 or 2) and to consider how the intervention could be adapted to work in their context.<br><br>Other elements of the intervention such as badges for workers and the flip chart will act as visual cues to remind workers of the training. *Badges will be worn during the working day and the flip charts will be kept in the pharmacy consulting room.*
**Item 9. Tailoring: If the intervention was planned to be personalised, titrated or adapted, then describe what, why, when, and how**	The content of the training will be adjusted according to advisor/pharmacy staff status. Pharmacy staff will attend only the first training session where engagement of smokers is discussed and advisors will attend both sessions.
**Item 10. Modifications: If the intervention was modified during the course of the study,**	*The intervention comprises an irreducible core of communication and behaviour change skills training, however participants will be encouraged to adapt other elements in ways that will work best in their own setting.*

**describe the changes (what, why, when, and how)**	No major change to the training element of the intervention is anticipated over the course of the trial.
**Item 11. How well (planned): If intervention adherence or fidelity was assessed, describe how and by whom, and if any strategies were used to maintain or improve fidelity, describe them**	Engagement of smokers will be assessed by actors visiting pharmacies and posing as smokers seeing help. Actors will make notes and complete a checklist after the visit.<br>Use of consultation techniques will be assessed by self audio-recording of consultations taking place in the pharmacy consulting room. These recordings will be transcribed and analysed qualitatively.<br>*Engagement and quit rates will be fed back to the advisors by email.*
**Item 12: How well (actual): If intervention adherence or fidelity was assessed, describe the extent to which the intervention was delivered as planned**	Not applicable.

*Features of the initial intervention are in plain text and final intervention in *italics*.
