# Supplementary material for: Equipping community pharmacy workers as agents for health behaviour change: developing and testing a theory-based smoking cessation intervention
Source: BMJ Open. 2017 Aug 11;7(8):e015637. doi: 10.1136/bmjopen-2016-015637 (PMC5724215; doi:10.1136/bmjopen-2016-015637)
Supplement: Author's manuscript [file bmjopen-2016-015637.draft_revisions.pdf]

BMJ Open

Equipping community pharmacy workers as agents for health behaviour change: developing and testing a theory-based intervention

Journal:	*BMJ Open*
Manuscript ID	bmjopen-2016-015637
Article Type:	Research
Date Submitted by the Author:	21-Dec-2016
Complete List of Authors:	Steed, Liz; Barts and The London School of Medicine and Dentistry, Centre for Primary Care and Public Health<br>Sohanpal, Ratna; Queen Marys School of Medicine and Dentistry, Centre for Primary Care and Public Health<br>James, Wai Yee; Barts and The London School of Medicine and Dentistry, Centre for Primary Care and Public Health<br>Rivas, Carol; University of Southamptom, Faculty of Health Sciences<br>Jumbe, Sandra; Barts and The London School of Medicine and Dentistry, Centre for Primary Care and Public Health<br>Chater, Angel; University College London, Centre for Behavioural Medicine<br>Todd, Adam; Durham University, Division of Pharmacy<br>Edwards, Elizabeth; Queen Mary University of London, Centre for Primary Care and Public Health, Blizard Institute<br>MacNeill, Virginia; University of Oxford<br>Macfarlane, Fraser; Barts and The London School of Medicine and Dentistry, Centre for Primary Care and Public Health<br>Greenhalgh, Trisha; University of Oxford, Nuffield Department of Primary Care Health Sciences<br>Griffiths, Chris; Barts and The London School of Medicine and Dentistry,<br>Eldridge, Sandra; Queen Mary University of London, Centre for Primary Care and Public Health<br>Taylor, Stephanie; Queen Mary University of London, Primary Care and Public Health<br>Walton, Robert; Barts and The London School of Medicine and Dentistry, Centre for Health Sciences
**Primary Subject Heading**:	Smoking and tobacco
Secondary Subject Heading:	Public health, Patient-centred medicine, Health services research
Keywords:	PRIMARY CARE, QUALITATIVE RESEARCH, PUBLIC HEALTH

SCHOLARONE™
Manuscripts

Equipping community pharmacy workers as agents for health behaviour change: developing and testing a theory-based intervention

Liz Steed[1]

Ratna Sohanpal[1]

Wai-Yee James[1]

Carol Rivas[2]

Sandra Jumbe[1]

Angel Chater[1 3]

Adam Todd[5]

Elizabeth Edwards[1]

Virginia Macneil[4]

Fraser Macfarlane[1]

Trisha Greenhalgh[4]

Chris Griffiths[1]

Sandra Eldridge[1]

Stephanie Taylor[1]

Robert Walton[1]

[1]Asthma UK Centre for Applied Research, Centre for Primary Care and Public Health, Barts and The London School of Medicine and Dentistry, Queen Mary University of London, London, UK

[2]Faculty of Health Sciences, University of Southampton, UK

[3]University of Bedfordshire

[4]University of Oxford

[5]Centre for Health and Inequalities Research (CHIR), University of Durham

Corresponding author:

Liz Steed

Asthma UK Centre for Applied Research, Centre for Primary Care and Public Health, Blizard Institute

Queen Mary University of London,

58 Turner Street, London

E1 2AB

Ph: +442078822492

Fax: +442078822552

Email: e.a.steed@qmul.ac.uk

For peer review only

For peer review only - http://bmjopen.bmj.com/site/about/guidelines.xhtml

ABSTRACT

Objective: To develop a complex intervention for community pharmacy staff to promote uptake of smoking cessation services and to increase quit rates.

Design: Following the Medical Research Council framework, we used a mixed methods approach to develop, pilot and then refine the intervention**.**

Methods: Phase One: we used information from qualitative studies in pharmacies, systematic literature reviews and the Capability Opportunity Motivation - Behaviour (COM-B) framework to inform design of the initial version of the intervention.

Phase Two: We then tested the acceptability of this intervention with smoking cessation advisors and assessed fidelity using actors who visited pharmacies posing as smokers, in a pilot study.

Phase Three: We reviewed the content and associated theory underpinning our intervention, taking account of the results of the earlier studies and a realist analysis of published literature. We then confirmed a logic model describing the intended operation of the intervention and used this model to refine the intervention and associated materials..

Setting: Eight community pharmacies in three inner east London boroughs.

Participants: 12 Stop Smoking Advisers

Intervention: Two, 150 minute, skills based training sessions focussed on communication and behaviour change skills with between session practice.

Results: The pilot study confirmed acceptability of the intervention and showed preliminary evidence of benefit, however organisational barriers tended to limit effective operation. The pilot data and realist review pointed to additional use of Diffusion of Innovations Theory to seat the intervention in the wider organisational context.

Conclusions: We have developed and refined an intervention to promote smoking cessation services in community pharmacies, which we now plan to evaluate in a randomised controlled trial.

265/300 words

Keywords: Smoking cessation, Community Pharmacies, Intervention development, Health Behaviour Change, Social Cognitive Theory, Self Determination Theory, Diffusion of Innovations Theory.

Word count (excluding figures and tables).

For peer review only - http://bmjopen.bmj.com/site/about/guidelines.xhtml

Strengths and limitations of this study

- We used a detailed mixed-methods approach aligned with MRC recommendations to develop a theory-driven intervention to promote smoking cessation in community pharmacies
- We used observational data together with theories of behaviour change to develop an initial version of the intervention which we piloted in pharmacies
- We used standardized simulated client methods to evaluate intervention fidelity by assessing real life pharmacy worker behaviour
- Following piloting and feasibility testing we refined the intervention using organisational theory to facilitate implementation
- We developed a detailed programme theory, specified through a logic model, to operationalize the hypothesised mechanisms of change

For peer review only

For peer review only - http://bmjopen.bmj.com/site/about/guidelines.xhtml

INTRODUCTION

Recent years have seen increasing use of community pharmacies for health behaviour change interventions. This shift in pharmacy practice in the UK is underpinned by Department of Health policy[1] and reflected in the drive towards Healthy Living Pharmacies.[2] Perhaps the best established of the new health promotion functions is smoking cessation, and community pharmacies now have a prominent place in delivery of NHS Stop Smoking Services, commissioned through a new contractual framework.[3] Whilst there is considerable potential for community pharmacies to support people in quitting, translation into effective practice may be less than optimal, since four-week quit rates are only 48% which is below the target 70%.[4 5] An important barrier may be a lack of awareness about the public health services now offered by community pharmacies.[6] Research also suggests that whilst pharmacy workers see a role for themselves in delivering interventions to change health behaviours, their level of confidence is low,[7] possibly related to lack of training,[8] and this may limit their effectiveness.

A number of studies have aimed to improve smoking cessation services in pharmacies through development of training sessions for pharmacists. These studies suggest some benefits including an increased level of smoking cessation counselling,[9,10] higher quit rates,[11,12] increased cost effectiveness[13] and sustained positive effects on pharmacist consulting behaviour.[12] A Cochrane review also shows that pharmacists could increase smoking cessation rates by providing a counselling and record-keeping support programme for their customers.[15]

Whilst previous interventions have shown benefits, these have not necessarily been translated into practice and their focus was primarily on the smoking cessation consultation itself rather than engagement into the stop smoking service. We therefore established The Smoking Treatment Optimisation in Pharmacies (STOP) programme, which aims to increase self-efficacy and motivation of pharmacy workers, promoting engagement of smokers into the NHS stop smoking service and optimising delivery of consultations. We aim to develop client-centred communication and behaviour change skills, thus enabling workers to facilitate health behaviour change in service users more effectively. The STOP intervention does not replace training provided by the National Centre for Smoking Cessation Training (NCSCT), but builds upon and reinforces many of the behaviour change techniques learnt.[16]

In developing the STOP intervention we aimed to build on results of previous studies by making the theoretical basis for the intervention more explicit and by taking into account the wider

For peer review only
For peer review only - http://bmjopen.bmj.com/site/about/guidelines.xhtml

organisational context in which the intervention is intended to operate. This paper reports: i) the process of developing the theory-based intervention; ii) evaluating acceptability to smoking cessation advisors and assessing fidelity in a pilot trial; iii) refinement of the intervention.

Phase One: Intervention Development

Methods

We followed the Medical Research Council guidelines for development of complex interventions[17 18] aiming to develop a programme theory with hypothesised mechanisms and predicates. We recognized that in order to change three distinct target behaviours of smokers namely a) engagement in stop smoking services, b) retention in the stop smoking service and c) sustained cessation, we needed to bring about fundamental change in pharmacy workers' consulting behaviour.

We based our initial work on the COM-B model (Capability, Opportunity, Motivation - Behaviour) which is a behaviour change framework used to guide development of behavioural interventions.[19 20] In this model behaviour is considered to be influenced by physical and psychological capacity to engage in an action, motivation towards the behaviour (including both conscious and habitual drivers) and by physical and social opportunity to perform the behaviour.[20] We considered each of these elements in relation to the three target behaviours to develop appropriate intervention materials and to plan training sessions.

The intervention was also informed by findings from five areas of work each described in detail below including: qualitative studies in local pharmacies (interviews and conversation analysis of audio recoded consultations); literature synthesis; realist review of smoking cessation interventions in community pharmacies; theoretical modelling; piloting in pharmacies. We also secured input from an expert professional advisory group comprising pharmacists (n=2), health psychologists (n=2), general practitioners (n=3), stop smoking experts (n=2), and trialists (n=2).

For peer review only - http://bmjopen.bmj.com/site/about/guidelines.xhtml

Results

Synthesis of prior literature

Our initial review of the literature on behaviour change interventions in community pharmacy [6 21 22] showed that such interventions could be successfully implemented and were most likely to be successful when supported by specific training. Further synthesis of the literature, provided information on practicalities such as length of training, with shorter durations (two hours) as effective, and potentially more acceptable, than longer training sessions.[23]

Qualitative Studies

Semi-structured interviews with pharmacy workers about their experiences of the smoking cessation service delivery were conducted and analysed using a framework approach[17] based on the COM-B model.[24] One key finding was that cessation advisors had preconceived ideas about which smokers were likely to join or to drop out of the service and made judgements about the client's likelihood of quitting. These judgements then influenced the time advisors would spend encouraging a smoker to join the service. In part this behaviour resulted from lack of confidence in skills to engage and to motivate smokers, particularly those not actively asking for help. There was also a concern about potentially challenging interactions with some smokers, for example smokers who particularly enjoyed smoking or those who did not see any harm in continuing to smoke. Advisors identified the need for extra training in client-centred consultation skills and for additional support to boost confidence. Some pharmacists felt that NHS remuneration for this activity was insufficient. Other pharmacy staff commented that if remuneration for pharmacists were increased, they could spend more time with smokers, which would result in more smokers joining and completing the service. There were some practical challenges such as insufficient time or resources that meant that advisors did not always seize opportunities for engagement and delivery of the service.

Conversation analysis was conducted on audio recordings of 16 pairs of smokers matched on gender, ethnicity, age and smoking intensity according to whether they were successful or unsuccessful in quitting. Quantitative and qualitative thematic analysis of consultation transcripts provided an overview of the data that helped us to determine aspects of the conversation within the consultation that might be associated with quitting or not quitting. Talk about the everyday experiences of smokers was common and apparently facilitated by a lack of social distance between advisors and their clients. This 'lifeworld' talk, when it was effectively used, enabled advisors to gain a better understanding of the smoker's perspective on the quit attempt. In this way advisors could give more effective support and facilitate more appropriate strategies to facilitate cessation.

For peer review only

For peer review only - http://bmjopen.bmj.com/site/about/guidelines.xhtml

Correspondingly, there was better alignment in reasons for quitting and relapsing between the advisors and smokers who successfully stopped smoking, compared to those who did not. It was apparent from the analysis that patient-centred talk was particularly likely to be to be omitted in deference to the 'voice of medicine' in non-quitters.[25] For example, the advisers would give detail about the physiology of smoking rather than addressing the social context of the smoking behaviour. This form of communication led to misalignment of advisor motivational strategies with smokers' reasons for quitting. Thus advisors often attempted to motivate clients with talk about financial savings, whereas smokers voiced health concerns more frequently as reasons for stopping smoking.

Developing a theory base for the initial intervention

Having identified the core issues to address, we reviewed which theories would best meet our needs. Specifically we matched constructs that we intended to target with theories that targeted these constructs, thus ensuring our intervention programme theory was underpinned with sound behavioural theory and had a clear hypothesized mechanism of action. To address capability we considered that Social Cognitive Theory[26] was relevant to target advisors' attitudes about benefits of improving smoking cessation services both for themselves and the smoker (Outcome expectancies). This theory was also relevant to enhancing advisor's confidence in engaging clients and delivering the smoking cessation service (Self Efficacy). To address motivation we considered that Self-Determination Theory[27] with its focus on intrinsic and extrinsic motivation would be useful, accounting for personal as well as external motivators. For example financial rewards were seen as important to pharmacists running a small business. To address Opportunity we set an in-practice discussion of how to translate learning into the specific pharmacy context as a task to complete before the second training session.

Overall, our aim in developing the initial version of the intervention was to develop more effective behaviour change skills in the smoking cessation advisors and to encourage smoker-centred care whilst avoiding a didactic biomedical approach.[28]

Expert advisory group

The group provided advice on each of the key elements of the intervention informing and overseeing the development process, both shaping the initial intervention and making adjustments to create the final version.

For peer review only

For peer review only - http://bmjopen.bmj.com/site/about/guidelines.xhtml

Initial intervention

Consensus within the expert group suggested that a two session face-to-face training programme targeting communication and behaviour change skills with homework tasks, social media support and a paper-based prompt tool should be the basis for the intervention. The social media support took the form of a Facebook page that included signposting to resources, links to videos on consulting styles and a discussion forum. Box 1 shows the content of the intervention with associated theory and behaviour change techniques.[29] Figure 1 shows the prompt tool developed as an aide memoir for use in smoking cessation consultations.

INSERT BOX 1 & FIGURE 1 ABOUT HERE

The pilot intervention was delivered jointly by a health psychologist (LS) and community pharmacist (DA) who was also a trained smoking cessation advisor and a National Centre for Smoking Cessation Training (NCSCT) smoking cessation trainer. We made videos with actors demonstrating effective communication skills and ways of asking questions with scripts informed by our audio recordings of real life consultations. We used role-plays, demonstration, brainstorming and problem-solving as teaching methods throughout the training sessions.

Where possible, the behaviour change techniques and communication skills in which advisors were being trained were modelled within the training sessions themselves. For example, advisors' personal motivations for implementing STOP were prompted in the same way that we intended them to elicit smokers' motivations to stop smoking. The training session on barriers and solutions for implementing the intervention used open-ended questions to elicit advisors' own solutions rather than simply offering solutions. These techniques mirror those that we expected the advisors to use with their clients.

Phase Two: Pilot testing of intervention acceptability and fidelity

Piloting delivery of the initial intervention and assessment of fidelity was carried out at the same time as piloting the study procedures necessary for the cluster randomised trial that will be used to evaluate the final version of the intervention. The process of intervention development was iterative so that the theoretical modelling and the results from piloting the intervention informed further data collection and analysis.

For peer review only - http://bmjopen.bmj.com/site/about/guidelines.xhtml

Methods

Study design

Participants

Any stop smoking advisors working in the London boroughs of Tower Hamlets or City and Hackney, and in a community pharmacy allocated to the intervention arm of the pilot trial, were eligible to participate in the STOP training. All advisors had completed NCSCT level two training (for further details of pilot trial see [30]). There were no restrictions on time elapsed since training as a stop smoking advisor. Advisors could be either pharmacists or other pharmacy workers such as counter assistants.

Acceptability of intervention

i) Attendance.

Details on attendance at each training session and any reasons given for non-attendance were documented.

ii) Qualitative assessment.

All participants who consented to participate in the trial, regardless of whether they attended any training sessions, were invited to attend a semi-structured interview one month after training. Views about the intervention and practical implementation were elicited. We also explored barriers and facilitators to attending training sessions and implementing the intervention. Interviews were conducted by a researcher independent of intervention delivery (RS) to minimize risk of social desirability responses. A framework analysis of the data [31] was conducted by RS.

Intervention fidelity and implementation in practice

We assessed fidelity of the intervention in terms of improving smoker engagement by using simulated clients, a method previously used for similar assessments in community pharmacy.[32] Two actors visited each pharmacy on different days, without prior knowledge of the pharmacy. The actors presented different clinical scenarios designed to give an opportunity for counter staff to engage them with the smoking cessation service. A matrix was used to ensure that two different case scenarios were presented to each pharmacy. Both actors were familiar with portraying simulated patients in clinical scenarios in Objective Structured Clinical Examinations (OSCE) for medical students.

For peer review only - http://bmjopen.bmj.com/site/about/guidelines.xhtml

Each actor completed a checklist following the visit which included items related to their interaction with the advisor e.g. whether a client would like to attend the smoking cessation service, general advisor communication style and use of study materials such as tar jars, stop smoking posters etc. Both actors underwent training before visiting pharmacies.

Self-Efficacy

An important hypothesized theoretical mechanism for change in advisor behaviour was through increased self-efficacy. We assessed this using a validated 0 - 5 point Likert scale, previously used in a similar context[33] such that higher ratings indicated greater self-efficacy. The scale was sent for self-completion one month after training to all advisors who attended.

RESULTS

Intervention Acceptability

All twelve stop smoking advisors in the intervention pharmacies agreed to participate in the training programme and gave informed consent. In total six advisors completed both training sessions (three at the group programme and three at a separate in-house training session). One participant withdrew after session one due to sickness and another because of low perceived relevance of the training sessions. Four participants did not attend any training for reasons detailed below.

Eight of the twelve advisors agreed to interview, four of whom had attended both training sessions, one had attended only session one and three had not attended any training. Six of those interviewed were pharmacists, one was a counter assistant and one a pharmacy technician (age range 24-52 years). Five interviewees were male. Themes emerging from the analysis of interview transcripts that were related to the training are presented in Table 1.

Table 1. Summary of qualitative findings from interviews with smoking cessation advisors related to intervention training

Theme	Illustrative Quotation
**Logistics/organisation of training**	*I think it was way out …I've never been there before, and because I close six thirty, …And I can't just leave at six thirty on the dot, I've got to tidy up things. So sometimes I don't leave here until maybe seven twenty or seven thirty,*
**Suggestions to improve the logistics of training**	*For us as pharmacists it's difficult because we need to have ourselves covered by another pharmacist, otherwise the business can't run. So unless there's compensation for*

For peer review only - http://bmjopen.bmj.com/site/about/guidelines.xhtml

	getting a locum pharmacists
**Training structure, content including the social media support element**	*Yeah it was great…got us involved…It was very informative, the way they actually made us do some play-acting to actually show a point to why they were doing that particular part of the talk. So that was all good.*<br><br>*to join (facebook) you would have to enrol as a company. So I don't think the boss really wanted Facebook as a company,… (Pharmacist)*
**Reflection on skills learnt**	*So I think this research should carry on for another year, not just six months. Because obviously we as practitioners need time…Because it's something new to us as well…There's more learning for us to do. It takes time for us to participate and engage.*<br>*Like when using the scales, when I try and engage customers I'll ask them on a scale of one to ten how ready are you to give up, one being not so ready and ten being very ready. So using those trigger questions they're very good, in that little book that we were given.*
**Improvement suggestions for training structure and content**	*I wonder whether there's an ability for the (other pharmacy) staff to be let off work maybe at a training session just for staff…or it could be a session where someone from your they can maybe…, come in for half an hour and do a similar thing to what we did. But not that in-depth that we were trained.*
**Application of learnt skills in practice and outcomes**	*When you come up to someone, say they came in and then they don't come back for a few weeks and then come back again, it's actually to keep the people in. So all the bits we've learnt additionally that we found now we've got a better success rate because people are coming back. Because we're saying…like sometimes if you talk to someone and you say oh well, you've smoked today, you shouldn't have really done that, but now we don't say that. We say look, this is a way to try around that, let's see what else we can try to stop you getting into that situation. That sort of thing. So we're using different ideas with them*

Logistics were identified as an important barrier to effective use of the training sessions, with participants reporting difficulty with both location (at a central training site) and timing (in the evenings 7pm-9.30pm). This was despite exploratory work that suggested evenings would be more convenient. Recommendations for venues closer to pharmacies and potential reimbursement to enable locum pharmacists to cover for pharmacist absence were made. The overall structure and content of the face-to-face training was acceptable and participants engaged well with activities, including role-play.

For peer review only - http://bmjopen.bmj.com/site/about/guidelines.xhtml

Only one advisor registered for the Facebook element of the intervention. Reasons for not engaging included not being allowed to use social media by managers, feeling uncomfortable with use of social media, and concern related to combining social and working lives. Participants reported that they felt able to use the newly learnt skills in practice and reported using specific techniques, for example confidence scales, to assist in consultations. Some participants, however, felt that it would take time for the new skills to become fully embedded in routine practice.

Fidelity assessment

Actor one visited all twelve intervention pharmacies, and actor two visited nine pharmacies. Table 2 shows ratings for the 18 items of the checklist. Actor two appeared to rate pharmacies more negatively than actor one and there was some inconsistency between responses for what might be expected to be objective items, for example the presence of a stop smoking poster and smoking prompts such as presence of a tar jar. Few pharmacy workers with whom the actors came into contact had attended the training (approximately 10%) as they were counter assistants not specifically trained in smoking cessation.

Table 2. Actor ratings for assessment of fidelity including display of smoking cessation materials, advisor communication skills and content of the consultation

	Actor 1		Actor 2	
	Yes	No	Yes	No
NHS Stop Smoking Service poster displayed	7	5	9	0
Audio-visual information about the NHS Stop Smoking Service	0	12	0	9
Leaflets about the NHS Stop Smoking Service	9	3	6	3
Smoking prompts e.g. tar jar	4	8	1	8
Were other clients observed being asked about smoking?	0	12	0	9
Good body language	6	6	6	3
Good listening skills	3	9	6	3
Use open questions	2	10	3	6

For peer review only - http://bmjopen.bmj.com/site/about/guidelines.xhtml

Was topic of smoking raised	0	12	0	9
Was smoking raised directly	1	11	0	9
Was smoking raised indirectly	0	12	0	9
Was client told there is a smoking cessation service	6	6	0	9
Was service highlighted as free aside from NRT	4	8	0	9
Was it service highlighted as delivered by experts	0	12	0	9
Was it highlighted 4x higher success rate with programme	2	10	0	9
Asked whether client want referral to the service	4	8	0	9
Assess whether client ready to change	2	10	0	9
Close by saying door is always open	5	7	0	9

Self-Efficacy Ratings

All 11 advisors completed the pre-training questionnaire and 10 completed questionnaires post-training. For pre-training, median score was 4 (range 3.5 to 4.6) and post training 4.5 (4.0-5.0).

Phase Three: Intervention Refinement

Key issues arising from the pilot and fidelity assessments that needed to be addressed in revising the STOP intervention were:-

- Ensuring that all pharmacy workers in contact with smokers were trained in smoker engagement (e.g. all counter assistants and not just smoking cessation advisors).
- Encouraging full attendance at the training sessions and display of study materials, financial reward (£30 cash per participant per session).
- Understanding organisational barriers to implementation of the intervention in community pharmacies and how these might be overcome.

In response to these issues we used realist review techniques to synthesise previous research on implementation of complex interventions for smoking cessation in community pharmacies, aiming to identify factors associated with success or failure of the intervention. The analysis took into account the environment in which the intervention was delivered and factors associated with the recipient,

For peer review only - http://bmjopen.bmj.com/site/about/guidelines.xhtml

together with features of the intervention itself that might influence effectiveness.[34] We gained insight into how specific interventions achieved their outcomes and, where there was an intervention that was only partially effective, the review indicated areas where aims had been achieved and the reasons why they might not have been achieved. The preliminary realist analysis identified four mechanisms upon which previously reported interventions had been operating: pharmacist identity; pharmacist capability; pharmacist motivation; stakeholder confidence. These mechanisms highlight specific attributes both of pharmacists and the wider organisational context that could potentially be exploited to enhance the effectiveness of pharmacy interventions for smoking cessation (Supplementary Table 1).[35]

We selected Diffusion of Innovations Theory (as adapted by Greenhalgh et al. for the study of organisational innovation in health care[36]) to seat the intervention within the organisational context of community pharmacies. This theory can be used to address how new methods of service delivery can be embedded and sustained in organisations. Amendments to the intervention according to the results of the initial piloting and review of theory are illustrated in Box 1 in italics. An overall description using the TIDieR framework[37] – a tool to facilitate explicit reporting of interventions – is presented in Supplementary Table 2.

The programme theory giving an overview of the intended operation of the intervention represented as a logic model[38] is shown in Figure 2.

INSERT FIGURE 2 ABOUT HERE

DISCUSSION

Statement of principal findings

We developed the STOP intervention in line with current Medical Research Council guidance for complex interventions, incorporating information from previous literature and results of our qualitative work with cessation advisors. We drew on theories about psychological aspects of human behaviour to develop our initial intervention. Interviews with pharmacy workers after the pilot study suggested that the face-to-face training was well received by those attending and that such training might improve self-efficacy in delivering smoking cessation interventions. However only six of 12

For peer review only - http://bmjopen.bmj.com/site/about/guidelines.xhtml

participants attended both training sessions and four individuals did not attend any sessions, suggesting a need for changing recruitment processes.

Our realist review[35] suggests that interventions to bring about behaviour change in pharmacy workers need to take into account role-identity, capability, motivation and the wider organisational context in which the intervention is operating. Behaviour change requires practitioner practice and continuing support with feedback[39] both to develop skills and to overcome anxieties about implementing a new form of practice. In the initial intervention this support was anticipated to be available through social media, but given that this was not readily accepted, a more traditional approach of follow-up sessions within practice is proposed for the revised intervention.

Social media is a relatively new phenomenon and currently seen as an area of great potential for many behavioural interventions,[40] however, our study identifies possible operational barriers that need to be considered. For example, health care professionals may not find it acceptable to blur the boundaries between professional and social life. For this form of intervention to be successful it may be necessary to give greater consideration to the motives and barriers towards using social media.[41]

Explicit attention needs to be given to the implementation of the intervention and integration with routine practice. Previous systematic reviews in other fields have similarly highlighted the importance of implementation.[42] In the revised STOP intervention we addressed effective implementation using Diffusion of Innovations theory. This theory aims to explain how new ideas spread in an organisational system and was useful in identifying key items that we needed to include in the intervention; for example allaying concerns before adoption and demonstrating that the intervention is not complex and fits well with the expanded role of community pharmacies. We also highlighted the need for flexibility in how the training sessions are delivered, for example at a central location or in individual pharmacies. The revised intervention training will be offered at different times of day and on different days of the week and those who are unable to attend will be offered on-site training.

Comparison with other studies

The pilot findings from STOP are in line with previous studies that found smoking cessation training acceptable to pharmacy workers.[9 10] The finding that logistics and organizational issues may act as a barrier to implementation is also in line with evidence that potentially effective interventions for

For peer review only - http://bmjopen.bmj.com/site/about/guidelines.xhtml

smoking cessation advisors have been developed but may not have changed quit rates or uptake of smoking cessation services.[43] The explicit application of organisational theory in development of the STOP intervention is an attempt to address this problem.

A realist review by our own team, undertaken in parallel with the fieldwork on this complex intervention and feeding into its final refinement, confirmed two key aspects of our empirical findings. First, that training in both factual knowledge and skills to enhance self-efficacy, informed by theories of behaviour change, improves the capacity and confidence (capability and motivation) of front-line pharmacy staff in supporting smoking cessation. Second, that organisational barriers (opportunity) may be significant even when individual motivation and capability are targeted. An additional finding from the realist analysis of the literature suggests that uptake and sustainability of pharmacy-based smoking cessation support depends on public and professional trust in the extended role of the pharmacist, which in turn depends on positive messages from the media and professional bodies. In the revised intervention we recommend support from local champions such as Healthy Living Leads which may partly address this issue.

Strengths and weaknesses

We developed a theoretical framework to underpin the intervention, drawing both from psychological theories related to behaviour change and organisational theory attempting to explain how interventions become incorporated into routine practice. Having established a framework, we translated this into behaviour change techniques to facilitate change in our three target behaviours: i) increased engagement of smokers; ii) retaining individuals in the stop smoking service; iii) improving quit rates. Our work builds upon the behaviour change techniques thought to be most effective for smoking cessation[16] but which may not be well implemented in routine practice.[43]

We based the intervention on our own qualitative work in community pharmacies, which helped us to gain an understanding of the detailed interactions between cessation advisors and their clients. Using our qualitative data from advisor interviews we were able to adjust the intervention according to the perceptions of the advisors about the nature and purpose of the smoking cessation service. In this way we aim to integrate the strong theoretical basis for the intervention with the real life pressures of routine work in community pharmacies.

Whilst the STOP intervention was developed in line with best practice for developing complex interventions, it is important to consider that both our pre-intervention development (qualitative

For peer review only - http://bmjopen.bmj.com/site/about/guidelines.xhtml

studies) and piloting drew upon the experience of pharmacies which were sufficiently engaged in the topic area to participate in a research study. There may be additional barriers to implementation for pharmacies that do not see smoking cessation as a priority. In addition there may be practical problems such as lack of a private consulting room, which could make implementation difficult in some pharmacies. The recent development of the Healthy Living Pharmacy framework[2] will mean that pharmacies are increasingly better equipped for smoking cessation and other health behaviour change tasks. It is therefore timely for pharmacy workers to be suitably trained in behaviour change skills.

Checks of Intervention fidelity are important when implementing a complex intervention.[43] In this study we used simulated clients to assess the degree to which core elements of the intervention were reflected in engagement behaviour of pharmacy staff. These methods have previously been used successfully in community pharmacies.[32 45 46] The simulated client methodology was informative in identifying areas where the intervention needed increased focus, for example the need to extend training to all community pharmacy workers. Whilst the simulated client visits provided a qualitative assessment of intervention fidelity and engagement behaviour, the scale of the pilot study did not allow quantitative assessment. In addition we found some discrepancy between actors in scoring apparently objective measures such as presence or absence of stop smoking posters. This discrepancy identifies a need for increased training of the actors and supports the suggestion that quantitative assessment of these factors may be important in the main trial. Obtaining adequate precision on engagement rates would require an increased number of scenarios and actors, which would increase costs.

In addition we found that the actors in the fidelity study often did not come into contact with trained pharmacy workers. This was because our pilot training was targeted at smoking cessation advisors and did not involve the counter assistants working in the pharmacies. Thus we identified a gap in referral of smokers from counter assistant to advisor, highlighting a need for involving counter assistants in the intervention.

Whilst we collected useful qualitative data from the advisor interviews, advisors' self-report of their experience of attending training sessions may be subject to social desirability responses. Nevertheless, the fact that a researcher independent from the intervention team conducted the interviews adds some validity to this assessment.

For peer review only - http://bmjopen.bmj.com/site/about/guidelines.xhtml

Studies developing smoking cessation interventions to date have mainly focussed on theories of individual behaviour change such as the transtheoretical model.[47] Whilst studies designed to test this approach show some benefit,[48] such theories tend not to take into account broader considerations of how the intervention might be implemented. It is a strength of the current study that we addressed organisational aspects likely to influence the effectiveness of the interventions. We are not aware of previous programmes in community pharmacies that have explicitly used organisational theory in addition to psychological theory to develop an intervention. Although some previous studies have compared different models of service delivery, these have not provided a theory-based exploration of the mechanisms by which the intervention brought about behaviour change in different settings.[49 50] As a result it is difficult to generalise from these trials because mechanisms may be operating differently in different community pharmacies.[51] A consideration of theoretical perspectives on the implementation of interventions to supplement psychological theories of behaviour change would seem prerequisite if the intervention is to work reliably in diverse settings. The final evaluation of the STOP intervention will include a process evaluation based on the mechanisms set out in the STOP logic model. This process evaluation may help to validate the STOP logic model and inform our plans for large-scale implementation should the intervention prove to be effective.

Implications for clinical practice and policy makers

We found that smoking cessation advisors were generally motivated to support their clients to change health behaviours and were receptive to additional skills training. It was apparent however that in order to engage smokers more effectively, a wider work force than stop smoking advisors needs to be trained. Nevertheless the results from our pilot studies provide initial evidence that STOP may be an acceptable and potentially effective intervention. [30]

Implications for future research

Whilst many health systems are expanding the role of community pharmacies into lifestyle management, there has been very little research on the effectiveness of pharmacy workers in their extended roles.[52] Since there are substantial economic implications for funding wider delivery of primary health care through pharmacies, health commissioners may need stronger evidence before decisions on allocation of funding are made. Such evidence will require large-scale studies to evaluate the effectiveness and cost effectiveness of changes in service delivery and further investigation into the organisational barriers to implementation.

For peer review only - http://bmjopen.bmj.com/site/about/guidelines.xhtml

Additional Information

The training and intervention materials, including the intervention manual, will be available upon request from the authors following completion of the evaluation phase of the STOP programme.

Acknowledgments The authors thank participants and their community pharmacies. This paper presents independent research funded by the National Institute for Health Research (NIHR) under its Programme Grants of Applied Research Programme. The views expressed are those of the authors and not necessarily those of the NHS, the NIHR or the Department of Health. We thank the independent members of the expert advisory group Professors Hayden Macrobbie and Rob Horne.

Contributors

This study is a sub-study of the NIHR funded STOP programme on which RW is chief investigator and CG, SE, ST and TG are co-investigators. LS led development of the initial version of the intervention and designed the fidelity assessment. RS, W-Y J, SJ, AC, CR, VM and EE recruited sites and collected and analysed qualitative and quantitative data. FM and TG conducted the realist analysis. AT and TG gave critical comments on the manuscript. LS and RW drafted the paper with input from all other authors. SJ assisted in refining the intervention. All authors have seen and approved the final manuscript.

Competing interests None.

Funding: NIHR Programme grant RP-PG-0609-10181

Participant consent Written informed consent obtained.

Ethics approval Ethical approval for the study was obtained from the NRES Committee South Central - Berkshire B (reference number: 13/SC/0189).

Provenance and peer review Not commissioned; externally peer reviewed

Data sharing statement

No additional data available.

For peer review only - http://bmjopen.bmj.com/site/about/guidelines.xhtml

Open Access This is an Open Access article distributed in accordance with the terms of the Creative Commons Attribution (CC BY 4.0) license, which permits others to distribute, remix, adapt and build upon this work, for commercial use, provided the original work is properly cited. See: http://creativecommons.org/licenses/by/4.0/

For peer review only

Figure legends

Figure 1. The Double Whammy paper based desk prompt

The desk prompt presents key messages from the training in a calendar format, displaying examples of how issues may be raised and addressed.

Figure 2. STOP programme theory

Logic model of the intended operation of the intervention.

For peer review only

For peer review only - http://bmjopen.bmj.com/site/about/guidelines.xhtml

References

1. Anonymous. Pharmacy in England. Building on strengths – delivering the future. In: Health, ed. London: H M Government, 2008.
2. Donovan GR, Paudyal V. England's Healthy Living Pharmacy (HLP) initiative: Facilitating the engagement of pharmacy support staff in public health. *Res Social Adm Pharm* 2016;12(2):281-92. doi: 10.1016/j.sapharm.2015.05.010
3. PSNC. Community Pharmacy Contractual Framework: Pharmaceutical Services Negotiating Committee; 2016 [Available from: http://psnc.org.uk/contract-it/the-pharmacy-contract/.
4. Lifestyle statistics Health and Social Care Information Centre. Statistics on NHS Stop Smoking Services: England, April 2012 – March 2013, 2013.
5. NHS Employers. Pharmacy-based stop smoking services: optimising commissioning. London: NHS Employers, 2009.
6. Lindsey L, Husband A, Steed L, et al. Helpful advice and hidden expertize: pharmacy users' experiences of community pharmacy accessibility. *J Public Health (Oxf)* 2016 doi: 10.1093/pubmed/fdw089
7. Eades C, Ferguson J, O'Carroll R. Public health in community pharmacy: A systematic review of pharmacist and consumer views. *BMC Public Health* 2011;11(1):582.
8. Jubraj B, Barnett NL, Grimes L, et al. Why we should understand the patient experience: clinical empathy and medicines optimisation. *Int J Pharm Pract* 2016;24(5):367-70. doi: 10.1111/ijpp.12268
9. Sinclair H, Bond C, Lennox, AS, et al. An evaluation of a training workshop for pharmacists based on the Stages of Change model of smoking cessation. *Health Education Journal* 1997;56:296-312.
10. Sinclair HK, Bond CM, Lennox AS, et al. Training pharmacists and pharmacy assistants in the stage-of-change model of smoking cessation: a randomised controlled trial in Scotland. *Tob Control* 1998;7(3):253-61.
11. Bock BC, Hudmon KS, Christian J, et al. A tailored intervention to support pharmacy-based counseling for smoking cessation. *Nicotine Tob Res* 2010;12(3):217-25. doi: 10.1093/ntr/ntp197
12. Maguire TA, McElnay JC, Drummond A. A randomized controlled trial of a smoking cessation intervention based in community pharmacies. *Addiction* 2001;96(2):325-31. doi: 10.1080/09652140020021062
13. Sinclair HK, Silcock J, Bond, CM, et al. The cost-effectiveness of intensive pharmaceutical intervention in assisting people to stop smoking. *The International Journal Of Pharmacy Practice* 1999;7:107-12.
14. Sinclair HK, Bond CM, Lennox AS. The long-term learning effect of training in stage of change for smoking cessation: a three-year follow up of community pharmacy staff's knowledge and attitudes. *International Journal of Pharmacy Practice* 1999;7(1):1-11.
15. Sinclair HK, Bond CM, Stead, LF et al. Community pharmacy personnel interventions for smoking cessation. *Cochrane Database of Systematic Reviews Issue 1 Art No: CD003698 DOI: 101002/14651858CD003698pub2* 2008:1-15.
16. West R, Walia A, Hyder N, et al. Behavior change techniques used by the English Stop Smoking Services and their associations with short-term quit outcomes. *Nicotine Tob Res* 2010;12(7):742-7. doi: 10.1093/ntr/ntq074
17. MRC. A framework for devleopment and evaluation of RCTs for complex interventions to improve health 2000 [Available from: https://www.mrc.ac.uk/documents/pdf/rcts-for-complex-interventions-to-improve-health/.
18. MRC. Developing and evaluating complex interventions: new guidance. 2008;https://www.mrc.ac.uk/documents/pdf/complex-interventions-guidance/

For peer review only - http://bmjopen.bmj.com/site/about/guidelines.xhtml

19. Michie S, Atkins L, West R. The Behaviour Change Wheel. A Guide to Designing Interventions: Silverback Publishing 2014.
20. Michie S, West R. The behaviour change wheel: A new method for characterising and designing behaviour change interventions. *Implementation Science* 2011;6
21. Steed L, Kassavou A, Madurasinghe VW, et al. Community pharmacy interventions for health promotion: effects on professional practice and health outcomes. *The Cochrane Library* 2014
22. Brown TJ, Todd A, O'Malley C, et al. Community pharmacy-delivered interventions for public health priorities: a systematic review of interventions for alcohol reduction, smoking cessation and weight management, including meta-analysis for smoking cessation. *BMJ Open* 2016;6(2):e009828. doi: 10.1136/bmjopen-2015-009828
23. Carson KV, Verbiest ME, Crone MR, et al. Training health professionals in smoking cessation. *Cochrane Database Syst Rev* 2012(5):CD000214. doi: 10.1002/14651858.CD000214.pub2
24. Sohanpal R, Rivas C, Steed L, et al. Understanding recruitment and retention in the NHS community pharmacy stop smoking service: perceptions of smoking cessation advisers. *BMJ Open* 2016;6(7):e010921. doi: 10.1136/bmjopen-2015-010921
25. Habermas J. The Theory of communicative action, reason and the rationalisation of society. : Heineman 1984.
26. Bandura A. Self-efficacy: Toward a unifying theory of behavioral change. *Psychological Review* 1977;84(2):191-215.
27. Deci EL, Ryan RM. Intrinsic motivation and self-determination in human behaviour. New York: Plenum 1985.
28. RCGP. Inquiry into Patient Centred Care in the 21st Century 2014 [14.8.16]. Available from: http://www.rcgp.org.uk/policy/rcgp-policy-areas/inquiry-into-patient-centred-care-in-the-21st-century.aspx.
29. Michie, S., Richardson, M., Johnston, M., Abraham, C., Francis, J., Hardeman, W., Wood, C. E. (2013). The behavior change technique taxonomy (v1) of 93 hierarchically clustered techniques: building an international consensus for the reporting of behavior change interventions. *Ann Behav Med, 46*(1), 81-95.
30. Madurasinghe VW, Sohanpal R, James W, et al. Smoking treatment optimisation in pharmacies (STOP): a cluster randomised pilot trial of a training intervention. *Pilot and Feasibility Studies*;(in press)
31. Richie, J and Spencer, L (1994), 'Qualitative data analysis for applied policy research', in Bryman and Burgess, eds., Analysing Qualitative Data, London: Routledge, p173-194.
32. Watson MC, Skelton JR, Bond CM, et al. Simulated patients in the community pharmacy setting. Using simulated patients to measure practice in the community pharmacy setting. *Pharm World Sci* 2004;26(1):32-7.
33. Brose LS, West R, Michie S, et al. Evaluation of Face-to-Face Courses in Behavioural Support for Stop Smoking Practitioners. *Journal of Smoking Cessation* 2012;7:25-30. doi: doi:10.1017/jsc.2012.6
34. Pawson R, Greenhalgh T, Harvey G, et al. Realist review--a new method of systematic review designed for complex policy interventions. *Journal of health services research & policy* 2005;10 Suppl 1:21-34. doi: 10.1258/1355819054308530 [published Online First: 2005/08/02]
35 .Greenhalgh T, McFarlane F, Steed L et al. What works for whom in pharmacist led smoking cessation services: A realist review BMC Medicine (2016) 14:209

For peer review only - http://bmjopen.bmj.com/site/about/guidelines.xhtml

36. Greenhalgh T, Robert G, Macfarlane F, et al. Diffusion of Innovations in Service Organizations: Systematic Review and Recommendations. *Milbank Quarterly* 2004;82(4):581-629. doi: 10.1111/j.0887-378X.2004.00325.x
37. Hoffmann TC, Glasziou PP, Boutron I, et al. Better reporting of interventions: template for intervention description and replication (TIDieR) checklist and guide. *BMJ* 2014;348 doi: 10.1136/bmj.g1687
38. FOUNDATION WKK. Using Logic Models to Bring Together Planning, Evaluation, and Action Logic Model Development Guide. One Michigan Avenue, East, Battle Creek, MI 49017-4012 USA. http://www.wkkf.org2006.
39. National Institute for Clinical Excellence Behaviour Change Individual Approaches. PH49. 2014 https://www.nice.org.uk/guidance/ph49/resources/behaviour-change-individual-approaches-1996366337989
40. Pagoto S, Waring ME, May CN, et al. Adapting Behavioral Interventions for Social Media Delivery. *J Med Internet Res* 2016;18(1):e24. doi: 10.2196/jmir.5086
41. Antheunis ML, Tates K, Nieboer TE. Patients' and health professionals' use of social media in health care: motives, barriers and expectations. Patient Educ Couns. 2013 Sep;92(3):426-31. doi: 10.1016/j.pec.2013.06.020.
42. Taylor S, Pinnock H, Epiphanou E, et al. A rapid synthesis of the evidence on interventions supporting self-management for people with long-term conditions: PRISMS. *Health Serv Deliv Res* 2014;2(53)
43. Lorencatto F, West R, Christopherson C, et al. Assessing fidelity of delivery of smoking cessation behavioural support in practice. *Implement Sci* 2013;8:40. doi: 10.1186/1748-5908-8-40
44. MRC. MRC PHRSN Process evaluation guidance final 2010 [Available from: https://www.mrc.ac.uk/documents/pdf/mrc-phsrn-process-evaluation-guidance-final/.
45. Watson MC, Cleland JA, Bond CM. Simulated patient visits with immediate feedback to improve the supply of over-the-counter medicines: a feasibility study. *Fam Pract* 2009;26(6):532-42. doi: 10.1093/fampra/cmp061
46. Inch J, Porteous T, Maskrey V, et al. It's not what you do it's the way that it's measured: quality assessment of minor ailment management in community pharmacies. *Int J Pharm Pract* 2016 doi: 10.1111/ijpp.12305
47. Prochaska JO, DiClemente CC. Stages and processes of self-change of smoking: toward an integrative model of change. *J Consult Clin Psychol* 1983;51(3):390-5.
48. Armitage CJ, Arden, M.A.,. How Useful Are the Stages of Change for Targeting Interventions? Randomized Test of a Brief Intervention to Reduce Smoking. *??* 2008;27(6):789-98.
49. Bauld L, Chesterman J, Ferguson J, et al. A comparison of the effectiveness of group-based and pharmacy-led smoking cessation treatment in Glasgow. *Addiction* 2009;104(2):308-16. doi: 10.1111/j.1360-0443.2008.02446.x
50. McEwen A, West R, McRobbie H. Effectiveness of specialist group treatment for smoking cessation vs. one-to-one treatment in primary care. *Addict Behav* 2006;31(9):1650-60. doi: 10.1016/j.addbeh.2005.12.014
51. Nkansah N, Mostovetsky O, Yu C, et al. Effect of outpatient pharmacists' non-dispensing roles on patient outcomes and prescribing patterns. *Cochrane Database Syst Rev* 2010(7):CD000336. doi: 10.1002/14651858.CD000336.pub2
52. Mossialos E, Naci H, Courtin E. Expanding the role of community pharmacists: policymaking in the absence of policy-relevant evidence? *International (Sweden / Canada / Belgium)* 2013;111(2):135-48. doi: 10.1016/j.healthpol.2013.04.003
53. Ryan RM, Deci EL. Self-Determination Theory and the Facilitation of Intrinsic Motivation, Social Development, and Well-Being. *American Psychologist* 2000;55(1):68-78.

For peer review only - http://bmjopen.bmj.com/site/about/guidelines.xhtml

Box 1. A detailed description of the STOP intervention showing underpinning theory and behaviour change techniques*

For peer review only

For peer review only - http://bmjopen.bmj.com/site/about/guidelines.xhtml

	Content	Theoretical Basis	Behaviour change techniques
Pharmacy visit	*Explain the study to pharmacist in charge or manager.* *Mention potential revenue stream from smoking cessation.* *Emphasise to staff how this fits well with their wider role in health promotion.* *Raise awareness in all staff in preparation for invitation to training.* *Emphasise intervention is brief.* *Address pre implementation concerns.* *Provide financial incentive for attending training (£30 per session, only received on completion of training)* *Emphasise backing from local and national opinion leaders and organisations (e.g. Local Pharmaceutical Committee, Royal Pharmaceutical Society, local CCGs and public health commissioners)*	*Adoption by individuals: concerns in preadoption stage (DIT)*<br><br>*The innovation: compatibility; relative advantage; low complexity (DIT)*<br><br>*Outer context: incentives (DIT)*<br><br>*Diffusion and dissemination: opinion leaders (DIT)*	*10.2 Material incentive (behaviour)*<br>*9.1 Credible source*<br>*1.2 Problem Solving*<br>*6.3 Information about others' approval*
**Training session one**			
**Introductions**	General orientation to the STOP programme, information on aims of training.<br><br>Include discussion of the impact of advisor behaviour on client stop smoking outcomes and health benefits to patients from stopping smoking.<br><br>*Communicate the advantages of the STOP intervention over usual practice, stress it's brief and show how it fits with overall 'pharmacy' identity.*	Outcome Expectancies (SCT)<br><br>*The innovation: relative advantage; compatibility; low complexity (DIT)*	9.1 Credible source<br><br>5.1 Information on health consequences of behaviour<br><br>15.1 Verbal persuasion about capability
**Topic 1: Why are we here?**	Exploration of motivation for helping smokers to quit.	Intrinsic and extrinsic	5.6 Information about emotional consequences

For peer review only - http://bmjopen.bmj.com/site/about/guidelines.xhtml

	Does engaging and supporting smokers' quit fit with role identity, any barriers? Encourage self perception as supporters and providers of health, how one will feel if help smokers quit. Emphasise the non-medication related, professional and public health aspects of the pharmacy role, promote a person-centred rather than product-centred ethos and foster a strong sense of professionalism	motivators (SDT)<br><br>*The innovation: compatibility (DIT)*	9.2 Pros and cons<br><br>6.3 Information about others approval<br><br>13.1 Identification of self as a role model
**Topic 2: Engaging Clients**	Group discussion of difficult/easy clients to engage – what are potential problems, solutions? Introduction of patient centred approach and basic communication skills including, rapport, listening, questioning. Role-play demonstration with senior pharmacist, participant practice. How to maximise opportunity with environmental resources e.g. tar jars. *Staff badges prompting client interaction*<br>Addressing pharmacy workers beliefs and attitudes e.g. prejudgement of success or failure.<br><br>*Emphasise predictable improved results,*	Self-efficacy (SCT)<br><br>Modeling (SCT)<br><br>Vicarious learning (SCT)<br><br>*The innovation: relative advantage; compatibility; low complexity (DIT)*	1.2 Problem solving,<br><br>4.1 Instruction on performance of behaviour,<br><br>6.1 Demonstration of behaviour,<br><br>8.1 Behavioural practice and rehearsal<br><br>7.1 Prompts and cues
**Topic 3: Is this the right time?**	Information on how to assess whether someone is ready to quit smoking. Using 1-10 scales to assess readiness to change and importance of change for the smoker. Role-play practice.<br><br>*Does engaging and supporting smokers' quit fit with role identity?*	Self-efficacy (SCT)<br><br>Self-regulation (SCT)<br><br>*The innovation: compatibility (DIT)*	4.1 Instruction how to perform behaviour<br><br>8.1 Behavioural practice and rehearsal
**Topic 4: Homework (revise NCSCT training, discuss how the intervention might be applied within your pharmacy)**	Encouragement to revise NSCSCT training in smoking cessation, including quizzes. Task to discuss as a pharmacy how might implement the STOP programme within their specific pharmacy – what	Self-efficacy (SCT)<br><br>Self-regulation (SCT)	1.1 Goal Setting (behaviour)<br>1.2 Problem Solving

For peer review only - http://bmjopen.bmj.com/site/about/guidelines.xhtml

	would be facilitators or barriers?<br><br>*Emphasise testing, trialling and adaptation to local circumstances.*	*The Innovation: trialabilty (DIT)*	
**Training session two**			
**Topic 1: Feedback & Reflections from Homework**	Discussion of homework. Key things learnt from completing NCSCT related training. Goal setting for filling in gaps<br><br>*Identification of organizational barriers, facilitators to implementing STOP in individual pharmacies. Facilitating action plans to implement STOP in their pharmacy.*<br><br>*Any further thoughts on how the intervention can be adapted to local circumstances?*	Self-regulation (SCT)<br><br>*The Innovation: fuzzy boundaries (DIT)*<br><br>*Adoption by individuals: concerns in preadoption stage (DIT)*	2.7 Feedback on outcome of behaviour<br>1.1 Goal Setting<br><br>1.2 Problem solving<br>1.4 Action Planning
**Topic 2: Challenge of Changing Behaviour & Maintaining a client-centred stance**	Brainstorming on what influences people to change behaviour – the role of beliefs, capability, opportunity in addition to knowledge.<br>How to elicit individuals' motivations, barriers and potential strategies to change behaviour versus offering solutions. Using 'What else questions'.<br>Understanding the 'non-smoker identity' and how to communicate to client<br>Demonstration & Role play<br><br>What makes this client-centred approach difficult, advantages, disadvantages, barriers and strategies to aid implementation	Outcome Expectancies (SCT)<br><br>Modelling (SCT)<br><br>Self-efficacy (SCT)	4.1 Instruction on how to perform behaviour<br><br>6.1 Demonstration of behaviour,<br><br>8.1 Behavioural practice and rehearsal<br><br>9.2 Pros and Cons<br><br>1.2 Problem Solving

For peer review only - http://bmjopen.bmj.com/site/about/guidelines.xhtml

**Topic 3: Planning a Quit & Dealing with Lapses**	Planning a quit – How to help people make a specific plan using a SMART approach. Setting own SMART goal. What to do if someone has a lapse, how to maintain support without excessive praise.<br>Watch and reflect on video of strong and weak consultations of quit planning. Discussion of how to talk about willpower and the role of the open door.<br>Demonstration & Role play	Modelling (SCT)<br><br>Self-efficacy (SCT)	4.1 Instruction on how to perform behaviour<br>1.1 Goal setting (behaviour)<br>6.1 Demonstration of behaviour<br>8.1 Behavioural practice and rehearsal
**Topic 4: Implementing STOP**	How to translate training to practice – barriers and solutions.<br>Use of prompts/cues including the Double Whammy (A desk top reminder with visual cues and example questions to ask) and *badges prompting client interaction.*<br>Introduction of social media support<br>*Using client wallet cards to remind patients of quit plans and motivators*	Self-regulation (SCT)<br>Intrinsic/Extrinsic Motivators (SDT)<br>*The Innovation: augmentation/support (DIT)*	4.1 Instruction on how to perform behaviour<br>1.2 Problem Solving<br>7.1 Prompts and Cues<br>3.1 Social support (practical and emotional)
**Topic 5: Goal Setting & Making a Commitment**	Develop a goal and elicit a commitment from participants to deliver STOP programme<br><br>Participants provided with a certificate for attending the training which is eligible for CPD points	Modelling (SCT)<br>Intrinsic/Extrinsic Motivators (SDT)<br>*Outer context: incentives (DIT)*	15.1 Verbal persuasion about capability<br>1.1 Goal Setting (behaviour)<br>1.9 Commitment<br>10.2 Material reward
Follow-Up Visit	*Promote adaptation of non-core elements of the intervention through a prompted pharmacy team*	*The innovation: trialability; Reinvention; fuzzy*	*1.4 Action planning*<br>*10.2 Material reward*

For peer review only

For peer review only - http://bmjopen.bmj.com/site/about/guidelines.xhtml

	meeting to discuss implementation of the intervention according to the needs of each individual pharmacy e.g. appointment of individual champions, monthly 'STOP' smoking days<br>*Provide financial reward for those who have completed intervention training*	*boundaries; champions (DIT)*	
	Be delivered in mixed groups of pharmacists and other pharmacy workers to promote cohesive working practices within the individual pharmacies.	*Implementation and routinisation: organizational structure (DIT)*	*3.2 Social support (practical)*

*Features of the initial intervention are in plain text and final intervention in *italics*.

For peer review only - http://bmjopen.bmj.com/site/about/guidelines.xhtml

31

For peer review only

Supplementary Table 1. Summary of preliminary findings from realist review

Mechanism by which the pharmacy smoking cessation service might be promoted	How the mechanism might be strengthened	Contextual influences
**Pharmacist identity**	• Strengthen 'pharmacy' identity by emphasising backing from professional bodies<br>• Promote non-medication and public health roles of the pharmacist<br>• Encourage patient-orientation rather than product-orientation<br>• Encourage a professional as opposed to 'technical' ethos	• Undergraduate education promotes these characteristics<br>• Professional bodies embrace extended role<br>• Policymakers recognise pharmacists as professionals
**Pharmacist capability**	• Strengthen knowledge base on health behavior change<br>• Consultation skills training<br>• Easily accessible educational sessions<br>• Change beliefs and attitudes, boosting self-efficacy in delivering the smoking cessation and encouraging belief that the intervention will be effective	• Quality, depth and breadth of training<br>• Training addresses skills and attitudes as well as knowledge<br>• Accessibility of training throughout professional life
**Pharmacist motivation**	• Present business arguments eg diversification of revenue streams, investment in space for financial returns<br>• Recognise training as continuing professional development	• Involvement of other pharmacies and pharmacists in health behavior change establishing a professional norm.<br>• Strong business model justifying investment in infrastructure<br>• Simple system for claiming payments
**Stakeholder confidence**	• Build confidence in the intervention from government, professional bodies (general practitioner and pharmacy), health commissioners.<br>• Change perceptions of patients and carers about the position of the pharmacist in health care system.	• Clear, positive messages in the media about the extended role<br>• Positive reaction to the role from other branches of primary care at national and local level

For peer review only - http://bmjopen.bmj.com/site/about/guidelines.xhtml

Supplementary Table 2. The intervention to be evaluated in the STOP randomised controlled trial described using the Template for Intervention Description and Replication (TIDieR) checklist.*

**Item 1. Brief name: Provide the name or a phrase that describes the intervention**	STOP - Service optimisation and communication skills training to strengthen the NHS smoking cessation service in community pharmacies.
**Item 2. Why: Describe any rationale, theory, or goal of the elements essential to the intervention**	We used the COM-B as a starting framework recognising the three behaviours of i) engaging smokers, ii) retaining smokers in the service and iii) smokers quit rates. For each we applied further theories including Social Cognitive theory [26] to underpin development of the intervention. The intervention aims to boost self-efficacy and self-regulation in the pharmacy staff to improve skills promoting recruitment and retention of service users and increasing quit success. We also drew upon Self Determination theory [27 50] to influence motivation which we see as important for the pharmacy staff to react to opportunities to promote the service and to create opportunities to discuss the service with potential clients.<br><br>We mapped the theoretical constructs onto specific behaviour change techniques using the behaviour change wheel and COM-B behaviour change model.[19 20] This model is increasingly used in development of behavioural interventions and has previously been used for smoking cessation. These behaviour change techniques were then embedded within the intervention.<br><br>*For implementation of the intervention we applied Diffusion of Innovations theory [34] which enabled us to seat the intervention in the complex organisational structures which we found both within and between community pharmacies.*<br><br>*The training stresses the advantages over usual practice in terms of revenue for the pharmacy and in building up the common good. The intervention is brief and fits in with usual practice and work flows and can be adapted in the context of each individual pharmacy. Concerns will be addressed about risks to profits – more important for the pharmacist/owners than for the staff. Backing from local opinion leaders will be emphasised (Hemant Patel, Secretary of the Local Pharmaceutical Committee) and we suggest that individual champions are appointed within each pharmacy perhaps also with a monthly 'STOP smoking day' to maintain the focus of attention.*

For peer review only - http://bmjopen.bmj.com/site/about/guidelines.xhtml

**Item 3. What (materials): Describe any physical or informational materials used in the intervention, including those provided to participants or used in intervention delivery or in training of intervention providers. Provide information on where the materials can be accessed**	Training material will be given as printed handouts in a study folder. The training sessions will involve role-plays and training videos – targeting engagement of smokers and optimising delivery of the stop smoking service aiming to increase retention and successful quitting. Attendees will be given a STOP flip chart (for desk) – with notes on key behaviour change techniques, 'Quit card' (can be kept in wallet) – to be given to smoker clients to reinforce smoking cessation strategies.<br><br>*The attendees will be given badges to wear in pharmacy as visual prompts to potential service users to ask about smoking cessation.*<br><br>*Social media will be used to perform a 'helpdesk' function using email and 'Whatsapp' to provide an easily accessible 'one-stop' platform to access study materials such as training videos. We will also signpost external smoking cessation resources using Whatsapp for example smoking cessation training websites, and latest cessation guidance.*<br><br>*All materials will be made available on a website to facilitate access by those not using social media.*
**Item 4. What (procedures): Describe each of the procedures, activities, and/or processes used in the intervention, including any enabling or support activities**	The training sessions will be face to face in a group of approximately 12 stop smoking advisors or pharmacy support staff. The STOP training will be incentivised for pharmacy staff by providing a cash amount of £30 to attend each session including travel expensed (£60 for both sessions) and a 'certificate of completion' to each staff member for attending and completing the training (see Appendix 2 and 3). The payment will be made only on completing the second session.
**Item 5. Who provided: For each category of intervention provider (for example, psychologist, nursing assistant), describe their expertise, background and any specific training given**	Training sessions will be facilitated by an experienced trainer/health psychologist and a community pharmacist, also a trainer. Skilled facilitation is needed for these groups, supported by the leader/co-leader structure and the pharmacists are then also available to answer practical questions about smoking cessation which we found arose frequently in the sessions that we conducted for the pilot study.
**Item 6. How: Describe the modes of delivery (such as face to face or by some other mechanism, such as internet or telephone) of the intervention and**	The face-to-face training will be delivered at varying times to accommodate the work routine of pharmacy staff.<br><br>See also Item 3 above for internet and social media resources.

For peer review only - http://bmjopen.bmj.com/site/about/guidelines.xhtml

**whether it was provided individually or in a group**	
**Item 7. Where: Describe the type(s) of location(s) where the intervention occurred, including any necessary infrastructure or relevant features**	The training will be delivered in training venues commonly used by pharmacy staff. Example of venues include the Tomlinson Centre in City and Hackney, Newham hospital or restaurants with separate meeting room in Newham. Venues will be chosen to be easily accessible to pharmacy workers after the pharmacy closes in the evening. Suggestions for venues will be taken from local Public Health commissioners and from the pharmacists themselves at recruitment.<br><br>Training sessions for pharmacy workers for other purposes are often held at restaurants where a meal or vouchers are provided and we will fit in with this model if possible if an appropriate meeting room is also available.
**Item 8. When and how much: Describe the number of times the intervention was delivered and over what period of time including the number of sessions, their schedule, and their duration, intensity or dose**	The training will comprise two sessions, each session of 2.5 hours, separated by two weeks. Pilot work suggested that longer sessions would not be more effective.<br><br>Tasks will be given at the end of the first session for advisors to complete NCSCT training (level 1 or 2) and to consider how the intervention could be adapted to work in their context.<br><br>Other elements of the intervention such as badges for workers and the flip chart will act as visual cues to remind workers of the training. *Badges will be worn during the working day and the flip charts will be kept in the pharmacy consulting room.*
**Item 9. Tailoring: If the intervention was planned to be personalised, titrated or adapted, then describe what, why, when, and how**	The content of the training will be adjusted according to advisor/pharmacy staff status. Pharmacy staff will attend only the first training session where engagement of smokers is discussed and advisors will attend both sessions.
**Item 10. Modifications: If the intervention was modified during the course of the study, describe the changes (what, why, when, and**	*The intervention comprises an irreducible core of communication and behaviour change skills training, however participants will be encouraged to adapt other elements in ways that will work best in their own setting.*

4

**how)**	No major change to the training element of the intervention is anticipated over the course of the trial.
**Item 11. How well (planned): If intervention adherence or fidelity was assessed, describe how and by whom, and if any strategies were used to maintain or improve fidelity, describe them**	Engagement of smokers will be assessed by actors visiting pharmacies and posing as smokers seeing help. Actors will make notes and complete a checklist after the visit.<br>Use of consultation techniques will be assessed by self audio-recording of consultations taking place in the pharmacy consulting room. These recordings will be transcribed and analysed qualitatively.<br>*Engagement and quit rates will be fed back to the advisors by email.*
**Item 12: How well (actual): If intervention adherence or fidelity was assessed, describe the extent to which the intervention was delivered as planned**	Not applicable.

*Features of the initial intervention are in plain text and final intervention in *italics*.

For peer review only
For peer review only - http://bmjopen.bmj.com/site/about/guidelines.xhtml

Figure 1. The Double Whammy desk prompt

STOP Smoking Treatment Optimisation in Pharmacies

W – WHO, WHAT, WHY?	W – WHEN?
H – HOW MANY?	H – HOW TO DO IT
A – ACTIONS TRIED	A – ADDITIONAL SUPPORT
M – MEDICATIONS	M – MONITORING
M – MOTIVATION	M – MAKE A COMMITMENT
Y – YOU THE NON SMOKER	Y – YOU CAN DO IT!

HELP PEOPLE QUIT WITH THE DOUBLE WHAMMY

Barts and The London School of Medicine and Dentistry www.smd.qmul.ac.uk

iew only

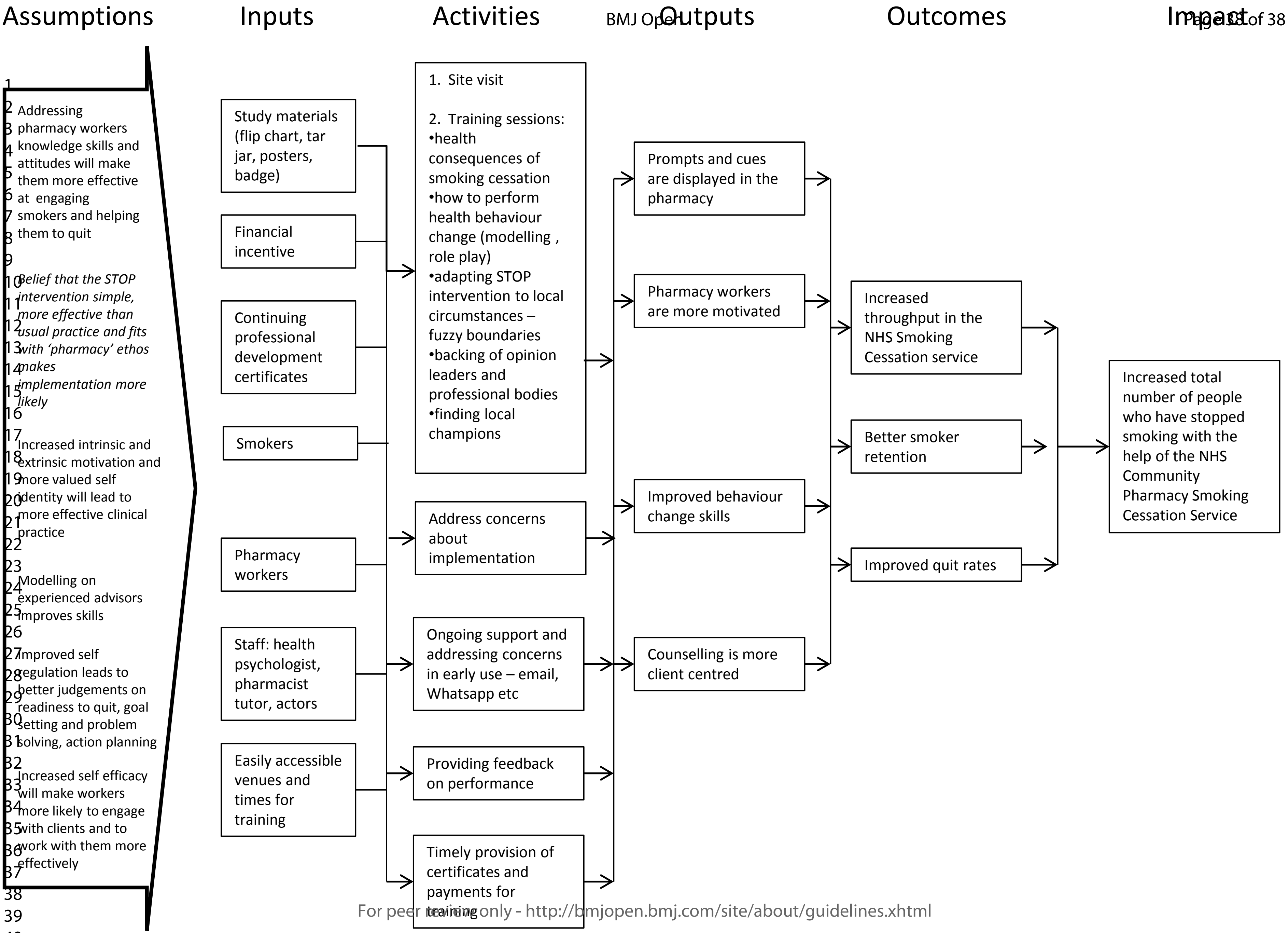
Assumptions
Inputs
Activities
Outputs
Outcomes
Impact
Addressing pharmacy workers knowledge skills and attitudes will make them more effective at engaging smokers and helping them to quit
Belief that the STOP intervention simple, more effective than usual practice and fits with 'pharmacy' ethos makes implementation more likely
Increased intrinsic and extrinsic motivation and more valued self identity will lead to more effective clinical practice
Modelling on experienced advisors improves skills
Improved self regulation leads to better judgements on readiness to quit, goal setting and problem solving, action planning
Increased self efficacy will make workers more likely to engage with clients and to work with them more effectively
Study materials (flip chart, tar jar, posters, badge)
Financial incentive
Continuing professional development certificates
Smokers
Pharmacy workers
Staff: health psychologist, pharmacist tutor, actors
Easily accessible venues and times for training
1. Site visit
2. Training sessions:
•health consequences of smoking cessation
•how to perform health behaviour change (modelling , role play)
•adapting STOP intervention to local circumstances – fuzzy boundaries
•backing of opinion leaders and professional bodies
•finding local champions
Address concerns about implementation
Ongoing support and addressing concerns in early use – email, Whatsapp etc
Providing feedback on performance
Timely provision of certificates and payments for training
Prompts and cues are displayed in the pharmacy
Pharmacy workers are more motivated
Improved behaviour change skills
Counselling is more client centred
Increased throughput in the NHS Smoking Cessation service
Better smoker retention
Improved quit rates
Increased total number of people who have stopped smoking with the help of the NHS Community Pharmacy Smoking Cessation Service

BMJ Open

Equipping community pharmacy workers as agents for health behaviour change: developing and testing a theory-based smoking cessation intervention

Journal:	*BMJ Open*
Manuscript ID	bmjopen-2016-015637.R1
Article Type:	Research
Date Submitted by the Author:	14-Mar-2017
Complete List of Authors:	Steed, Liz; Barts and The London School of Medicine and Dentistry, Centre for Primary Care and Public Health<br>Sohanpal, Ratna; Queen Marys School of Medicine and Dentistry, Centre for Primary Care and Public Health<br>James, Wai Yee; Barts and The London School of Medicine and Dentistry, Centre for Primary Care and Public Health<br>Rivas, Carol; University of Southamptom, Faculty of Health Sciences<br>Jumbe, Sandra; Barts and The London School of Medicine and Dentistry, Centre for Primary Care and Public Health<br>Chater, Angel; University College London, Centre for Behavioural Medicine<br>Todd, Adam; Durham University, Division of Pharmacy<br>Edwards, Elizabeth; Queen Mary University of London, Centre for Primary Care and Public Health, Blizard Institute<br>MacNeill, Virginia; University of Oxford<br>Macfarlane, Fraser; Barts and The London School of Medicine and Dentistry, Centre for Primary Care and Public Health<br>Greenhalgh, Trisha; University of Oxford, Nuffield Department of Primary Care Health Sciences<br>Griffiths, Chris; Barts and The London School of Medicine and Dentistry,<br>Eldridge, Sandra; Queen Mary University of London, Centre for Primary Care and Public Health<br>Taylor, Stephanie; Queen Mary University of London, Primary Care and Public Health<br>Walton, Robert; Barts and The London School of Medicine and Dentistry, Centre for Health Sciences
**Primary Subject Heading**:	Smoking and tobacco
Secondary Subject Heading:	Public health, Patient-centred medicine, Health services research
Keywords:	PRIMARY CARE, QUALITATIVE RESEARCH, PUBLIC HEALTH

SCHOLARONE™
Manuscripts

Equipping community pharmacy workers as agents for health behaviour change: developing and testing a theory-based smoking cessation intervention

Liz Steed[1]

Ratna Sohanpal[1]

Wai-Yee James[1]

Carol Rivas[2]

Sandra Jumbe[1]

Angel Chater[1 3]

Adam Todd[5]

Elizabeth Edwards[1]

Virginia Macneil[4]

Fraser Macfarlane[1]

Trisha Greenhalgh[4]

Chris Griffiths[1]

Sandra Eldridge[1]

Stephanie Taylor[1]

Robert Walton[1]

[1]Asthma UK Centre for Applied Research, Centre for Primary Care and Public Health, Barts and The London School of Medicine and Dentistry, Queen Mary University of London, London, UK

[2]Faculty of Health Sciences, University of Southampton, UK

[3]University of Bedfordshire

[4]University of Oxford

[5]Centre for Health and Inequalities Research (CHIR), University of Durham

Corresponding author:

Liz Steed

Asthma UK Centre for Applied Research, Centre for Primary Care and Public Health, Blizard Institute

Queen Mary University of London,

58 Turner Street, London

E1 2AB

Ph: +442078822492

Fax: +442078822552

Email: e.a.steed@qmul.ac.uk

For peer review only

ABSTRACT

Objective: To develop a complex intervention for community pharmacy staff to promote uptake of smoking cessation services and to increase quit rates.

Design: Following the Medical Research Council framework, we used a mixed methods approach to develop, pilot and then refine the intervention**.**

Methods: Phase One: we used information from qualitative studies in pharmacies, systematic literature reviews and the Capability Opportunity Motivation - Behaviour (COM-B) framework to inform design of the initial version of the intervention.

Phase Two: We then tested the acceptability of this intervention with smoking cessation advisors and assessed fidelity using actors who visited pharmacies posing as smokers, in a pilot study.

Phase Three: We reviewed the content and associated theory underpinning our intervention, taking account of the results of the earlier studies and a realist analysis of published literature. We then confirmed a logic model describing the intended operation of the intervention and used this model to refine the intervention and associated materials..

Setting: Eight community pharmacies in three inner east London boroughs.

Participants: 12 Stop Smoking Advisers

Intervention: Two, 150 minute, skills based training sessions focussed on communication and behaviour change skills with between session practice.

Results: The pilot study confirmed acceptability of the intervention and showed preliminary evidence of benefit, however organisational barriers tended to limit effective operation. The pilot data and realist review pointed to additional use of Diffusion of Innovations Theory to seat the intervention in the wider organisational context.

Conclusions: We have developed and refined an intervention to promote smoking cessation services in community pharmacies, which we now plan to evaluate in a randomised controlled trial.

265/300 words

Keywords: Smoking cessation, Community Pharmacies, Intervention development, Health Behaviour Change, Social Cognitive Theory, Self Determination Theory, Diffusion of Innovations Theory.

Word count (excluding figures and tables).

For peer review only

Strengths and limitations of this study

- We used a detailed mixed-methods approach aligned with MRC recommendations to develop a theory-driven intervention to promote smoking cessation in community pharmacies
- We used observational data together with theories of behaviour change to develop an initial version of the intervention which we piloted in pharmacies
- We used standardized simulated client methods to evaluate intervention fidelity by assessing real life pharmacy worker behaviour
- Following piloting and feasibility testing we refined the intervention using organisational theory to facilitate implementation
- We developed a detailed programme theory, specified through a logic model, to operationalize the hypothesised mechanisms of change

For peer review only

INTRODUCTION

Recent years have seen increasing use of community pharmacies for health behaviour change interventions. This shift in pharmacy practice in the UK is underpinned by Department of Health policy[1] and reflected in the drive towards Healthy Living Pharmacies.[2] Perhaps the best established of the new health promotion functions is smoking cessation, and community pharmacies now have a prominent place in delivery of NHS Stop Smoking Services, commissioned through a new contractual framework.[3] Whilst there is considerable potential for community pharmacies to support people in quitting, translation into effective practice may be less than optimal, since four-week quit rates are only 48% which is below the target 70%.[4 5] An important barrier may be a lack of awareness about the public health services now offered by community pharmacies.[6] Research also suggests that whilst pharmacy workers see a role for themselves in delivering interventions to change health behaviours, their level of confidence is low,[7] possibly related to lack of training,[8] and this may limit their effectiveness.

A number of studies have aimed to improve smoking cessation services in pharmacies through development of training sessions for pharmacists. These studies suggest some benefits including an increased level of smoking cessation counselling,[9,10] higher quit rates,[11,12] increased cost effectiveness[13] and sustained positive effects on pharmacist consulting behaviour.[12,14] A Cochrane review also shows that pharmacists could increase smoking cessation rates by providing a counselling and record-keeping support programme for their customers.[15]

Whilst previous interventions have shown benefits, these have not necessarily been translated into practice and their focus was primarily on the smoking cessation consultation itself rather than engagement into the stop smoking service. We therefore established the Smoking Treatment Optimisation in Pharmacies (STOP) programme, which aims to increase self-efficacy and motivation of pharmacy workers, promoting engagement of smokers into the NHS stop smoking service and optimising delivery of consultations. We aim to develop client-centred communication and behaviour change skills, thus enabling workers to facilitate health behaviour change in service users more effectively. The STOP intervention does not replace training provided by the National Centre for Smoking Cessation Training (NCSCT), but builds upon and reinforces many of the behaviour change techniques learnt.[16]

In developing the STOP intervention we aimed to build on results of previous studies by making the theoretical basis for the intervention more explicit and by taking into account the wider

For peer review only

organisational context in which the intervention is intended to operate. This paper reports: i) the process of developing the theory-based intervention; ii) evaluating acceptability to smoking cessation advisors and assessing fidelity in a pilot trial; iii) refinement of the intervention.

Phase One: Intervention Development

Methods

We followed the Medical Research Council guidelines for development of complex interventions[17 18] aiming to develop a programme theory with hypothesised mechanisms and predicates. We recognized that in order to change three distinct target behaviours of smokers namely a) engagement in stop smoking services, b) retention in the stop smoking service and c) sustained cessation, we needed to bring about fundamental change in pharmacy workers' consulting behaviour.

We based our initial work on the COM-B model (Capability, Opportunity, Motivation - Behaviour) which is a behaviour change framework used to guide development of behavioural interventions.[19 20] In this model behaviour is considered to be influenced by physical and psychological capacity to engage in an action, motivation towards the behaviour (including both conscious and habitual drivers) and by physical and social opportunity to perform the behaviour.[20] We considered each of these elements in relation to the three target behaviours to develop appropriate intervention materials and to plan training sessions.

The intervention was also informed by findings from five areas of work each described in detail below including: qualitative studies in local pharmacies (interviews and conversation analysis of audio recoded consultations); literature synthesis; realist review of smoking cessation interventions in community pharmacies; theoretical modelling; piloting in pharmacies. We also secured input from an expert professional advisory group comprising pharmacists (n=2), health psychologists (n=2), general practitioners (n=3), stop smoking experts (n=2), and trialists (n=2).

For peer review only

Results

Synthesis of prior literature

Our initial review of the literature on behaviour change interventions in community pharmacy [6 21 22] showed that such interventions could be successfully implemented and were most likely to be successful when supported by specific training. Further synthesis of the literature, provided information on practicalities such as length of training, with shorter durations (two hours) as effective, and potentially more acceptable, than longer training sessions.[23]

Qualitative Studies

Semi-structured interviews with pharmacy workers about their experiences of the smoking cessation service delivery were conducted and analysed using a framework approach[17] based on the COM-B model.[24] One key finding was that cessation advisors had preconceived ideas about which smokers were likely to join or to drop out of the service and made judgements about the client's likelihood of quitting. These judgements then influenced the time advisors would spend encouraging a smoker to join the service. In part this behaviour resulted from lack of confidence in skills to engage and to motivate smokers, particularly those not actively asking for help. There was also a concern about potentially challenging interactions with some smokers, for example smokers who particularly enjoyed smoking or those who did not see any harm in continuing to smoke. Advisors identified the need for extra training in client-centred consultation skills and for additional support to boost confidence. Some pharmacists felt that NHS remuneration for this activity was insufficient. Other pharmacy staff commented that if remuneration for pharmacists were increased, they could spend more time with smokers, which would result in more smokers joining and completing the service. There were some practical challenges such as insufficient time or resources that meant that advisors did not always seize opportunities for engagement and delivery of the service.

Conversation analysis was conducted on audio recordings of 16 pairs of smokers matched on gender, ethnicity, age and smoking intensity according to whether they were successful or unsuccessful in quitting. Quantitative and qualitative thematic analysis of consultation transcripts provided an overview of the data that helped us to determine aspects of the conversation within the consultation that might be associated with quitting or not quitting. Talk about the everyday experiences of smokers was common and apparently facilitated by a lack of social distance between advisors and their clients. This 'lifeworld' talk, when it was effectively used, enabled advisors to gain a better understanding of the smoker's perspective on the quit attempt. In this way advisors could give more effective support and facilitate more appropriate strategies to facilitate cessation.

For peer review only

Correspondingly, there was better alignment in reasons for quitting and relapsing between the advisors and smokers who successfully stopped smoking, compared to those who did not. It was apparent from the analysis that patient-centred talk was particularly likely to be to be omitted in deference to the 'voice of medicine' in non-quitters.[25] For example, the advisers would give detail about the physiology of smoking rather than addressing the social context of the smoking behaviour. This form of communication led to misalignment of advisor motivational strategies with smokers' reasons for quitting. Thus advisors often attempted to motivate clients with talk about financial savings, whereas smokers voiced health concerns more frequently as reasons for stopping smoking.

Developing a theory base for the initial intervention

Having identified the core issues to address, we reviewed which theories would best meet our needs. Specifically we matched constructs that we intended to target with theories that targeted these constructs, thus ensuring our intervention programme theory was underpinned with sound behavioural theory and had a clear hypothesized mechanism of action. To address capability we considered that Social Cognitive Theory[26] was relevant to target advisors' attitudes about benefits of improving smoking cessation services both for themselves and the smoker (Outcome expectancies). This theory was also relevant to enhancing advisor's confidence in engaging clients and delivering the smoking cessation service (Self Efficacy). To address motivation we considered that Self-Determination Theory[27] with its focus on intrinsic and extrinsic motivation would be useful, accounting for personal as well as external motivators. For example financial rewards were seen as important to pharmacists running a small business. To address Opportunity we set an in-practice discussion of how to translate learning into the specific pharmacy context as a task to complete before the second training session.

Overall, our aim in developing the initial version of the intervention was to develop more effective behaviour change skills in the smoking cessation advisors and to encourage smoker-centred care whilst avoiding a didactic biomedical approach.[28]

Expert advisory group

The group provided advice on each of the key elements of the intervention informing and overseeing the development process, both shaping the initial intervention and making adjustments to create the final version.

For peer review only

Initial intervention

Consensus within the expert group suggested that a two session face-to-face training programme targeting communication and behaviour change skills with homework tasks, social media support and a paper-based prompt tool should be the basis for the intervention. The social media support took the form of a Facebook page that included signposting to resources, links to videos on consulting styles and a discussion forum. Box 1 shows the content of the intervention with associated theory and behaviour change techniques.[29] Figure 1 shows the prompt tool developed as an aide memoir for use in smoking cessation consultations.

For peer review only

Box 1. A detailed description of the STOP intervention showing underpinning theory and behaviour change techniques*

	Content	Theoretical Basis	Behaviour change techniques
Pharmacy visit	*Explain the study to pharmacist in charge or manager.*<br>*Mention potential revenue stream from smoking cessation.*<br>*Emphasise to staff how this fits well with their wider role in health promotion.*<br>*Raise awareness in all staff in preparation for invitation to training.*<br>*Emphasise intervention is brief.*<br>*Address pre implementation concerns.*<br>*Provide financial incentive for attending training (£30 per session, only received on completion of training)*<br>*Emphasise backing from local and national opinion leaders and organisations (e.g. Local Pharmaceutical Committee, Royal Pharmaceutical Society, local CCGs and public health commissioners)*	*Adoption by individuals: concerns in preadoption stage (DIT)*<br><br>*The innovation: compatibility;*<br>*relative advantage; low complexity (DIT)*<br><br>*Outer context: incentives (DIT)*<br><br>*Diffusion and dissemination: opinion leaders (DIT)*	*10.2 Material incentive (behaviour)*<br>*9.1 Credible source*<br>*1.2 Problem Solving*<br>*6.3 Information about others' approval*
**Training session one**			
**Introductions**	General orientation to the STOP programme, information on aims of training.<br><br>Include discussion of the impact of advisor behaviour on client stop smoking outcomes and health benefits to patients from stopping smoking.<br><br>*Communicate the advantages of the STOP intervention over usual practice, stress it's brief and show how it fits with overall 'pharmacy' identity.*	Outcome Expectancies (SCT)<br><br>*The innovation: relative advantage; compatibility; low complexity (DIT)*	9.1 Credible source<br><br>5.1 Information on health consequences of behaviour<br><br>15.1 Verbal persuasion about capability

For peer review only

**Topic 1: Why are we here?**	Exploration of motivation for helping smokers to quit.<br><br>Does engaging and supporting smokers' quit fit with role identity, any barriers? Encourage self perception as supporters and providers of health, how one will feel if help smokers quit. Emphasise the non-medication related, professional and public health aspects of the pharmacy role, promote a person-centred rather than product-centred ethos and foster a strong sense of professionalism	Intrinsic and extrinsic motivators (SDT)<br><br>*The innovation: compatibility (DIT)*	5.6 Information about emotional consequences<br><br>9.2 Pros and cons<br><br>6.3 Information about others approval<br><br>13.1 Identification of self as a role model
**Topic 2: Engaging Clients**	Group discussion of difficult/easy clients to engage – what are potential problems, solutions? Introduction of patient centred approach and basic communication skills including, rapport, listening, questioning. Role-play demonstration with senior pharmacist, participant practice. How to maximise opportunity with environmental resources e.g. tar jars. *Staff badges prompting client interaction*<br>Addressing pharmacy workers beliefs and attitudes e.g. prejudgement of success or failure.<br><br>*Emphasise predictable improved results,*	Self-efficacy (SCT)<br><br>Modeling (SCT)<br><br>Vicarious learning (SCT)<br><br>*The innovation: relative advantage; compatibility; low complexity (DIT)*	1.2 Problem solving,<br><br>4.1 Instruction on performance of behaviour,<br><br>6.1 Demonstration of behaviour,<br><br>8.1 Behavioural practice and rehearsal<br><br>7.1 Prompts and cues
**Topic 3: Is this the right time?**	Information on how to assess whether someone is ready to quit smoking. Using 1-10 scales to assess readiness to change and importance of change for the smoker. Role-play practice.<br><br>*Does engaging and supporting smokers' quit fit with role identity?*	Self-efficacy (SCT)<br><br>Self-regulation (SCT)<br><br>*The innovation: compatibility (DIT)*	4.1 Instruction how to perform behaviour<br><br>8.1 Behavioural practice and rehearsal
**Topic 4: Homework (revise NCSCT training, discuss how the intervention**	Encouragement to revise NSCSCT training in smoking cessation, including quizzes. Task to discuss as a	Self-efficacy (SCT)	1.1 Goal Setting (behaviour)<br>1.2 Problem Solving

**might be applied within your pharmacy)**	pharmacy how might implement the STOP programme within their specific pharmacy – what would be facilitators or barriers?<br><br>*Emphasise testing, trialling and adaptation to local circumstances.*	Self-regulation (SCT)<br><br>*The Innovation: trialabilty (DIT)*	
**Training session two**			
**Topic 1: Feedback & Reflections from Homework**	Discussion of homework. Key things learnt from completing NCSCT related training. Goal setting for filling in gaps<br><br>*Identification of organizational barriers, facilitators to implementing STOP in individual pharmacies. Facilitating action plans to implement STOP in their pharmacy.*<br><br>*Any further thoughts on how the intervention can be adapted to local circumstances?*	Self-regulation (SCT)<br><br>*The Innovation: fuzzy boundaries (DIT)*<br><br>*Adoption by individuals: concerns in preadoption stage (DIT)*	2.7 Feedback on outcome of behaviour<br>1.1 Goal Setting<br><br>1.2 Problem solving<br>1.4 Action Planning
**Topic 2: Challenge of Changing Behaviour & Maintaining a client-centred stance**	Brainstorming on what influences people to change behaviour – the role of beliefs, capability, opportunity in addition to knowledge.<br>How to elicit individuals' motivations, barriers and potential strategies to change behaviour versus offering solutions. Using 'What else questions'.<br>Understanding the 'non-smoker identity' and how to communicate to client<br>Demonstration & Role play<br><br>What makes this client-centred approach difficult, advantages, disadvantages, barriers and	Outcome Expectancies (SCT)<br><br>Modelling (SCT)<br><br>Self-efficacy (SCT)	4.1 Instruction on how to perform behaviour<br><br>6.1 Demonstration of behaviour,<br><br>8.1 Behavioural practice and rehearsal<br><br>9.2 Pros and Cons<br><br>1.2 Problem Solving

	strategies to aid implementation		
**Topic 3: Planning a Quit & Dealing with Lapses**	Planning a quit – How to help people make a specific plan using a SMART approach. Setting own SMART goal. What to do if someone has a lapse, how to maintain support without excessive praise.<br>Watch and reflect on video of strong and weak consultations of quit planning. Discussion of how to talk about willpower and the role of the open door.<br>Demonstration & Role play	Modelling (SCT)<br><br>Self-efficacy (SCT)	4.1 Instruction on how to perform behaviour<br>1.1 Goal setting (behaviour)<br>6.1 Demonstration of behaviour<br>8.1 Behavioural practice and rehearsal
**Topic 4: Implementing STOP**	How to translate training to practice – barriers and solutions.<br>Use of prompts/cues including the Double Whammy (A desk top reminder with visual cues and example questions to ask) and *badges prompting client interaction.*<br>Introduction of social media support<br>*Using client wallet cards to remind patients of quit plans and motivators*	Self-regulation (SCT)<br>Intrinsic/Extrinsic Motivators (SDT)<br>*The Innovation: augmentation/support (DIT)*	4.1 Instruction on how to perform behaviour<br>1.2 Problem Solving<br>7.1 Prompts and Cues<br>3.1 Social support (practical and emotional)
**Topic 5: Goal Setting & Making a Commitment**	Develop a goal and elicit a commitment from participants to deliver STOP programme<br><br>Participants provided with a certificate for attending the training which is eligible for CPD points	Modelling (SCT)<br>Intrinsic/Extrinsic Motivators (SDT)<br>*Outer context: incentives (DIT)*	15.1 Verbal persuasion about capability<br>1.1 Goal Setting (behaviour)<br>1.9 Commitment<br>10.2 Material reward
Follow-Up Visit	*Promote adaptation of non-core elements of the intervention through a prompted pharmacy team*	*The innovation: trialability; Reinvention; fuzzy*	*1.4 Action planning*<br>*10.2 Material reward*

For peer review only

	meeting to discuss implementation of the intervention according to the needs of each individual pharmacy e.g. appointment of individual champions, monthly 'STOP' smoking days<br>*Provide financial reward for those who have completed intervention training*	*boundaries; champions (DIT)*	
	Be delivered in mixed groups of pharmacists and other pharmacy workers to promote cohesive working practices within the individual pharmacies.	*Implementation and routinisation: organizational structure (DIT)*	*3.2 Social support (practical)*

*Features of the initial intervention are in plain text and final intervention in *italics*.

For peer review only

INSERT FIGURE 1 ABOUT HERE

The pilot intervention was delivered jointly by a health psychologist (LS) and community pharmacist (DA) who was also a trained smoking cessation advisor and a National Centre for Smoking Cessation Training (NCSCT) smoking cessation trainer. We made videos with actors demonstrating effective communication skills and ways of asking questions with scripts informed by our audio recordings of real life consultations. We used role-plays, demonstration, brainstorming and problem-solving as teaching methods throughout the training sessions.

Where possible, the behaviour change techniques and communication skills in which advisors were being trained were modelled within the training sessions themselves. For example, advisors' personal motivations for implementing STOP were prompted in the same way that we intended them to elicit smokers' motivations to stop smoking. The training session on barriers and solutions for implementing the intervention used open-ended questions to elicit advisors' own solutions rather than simply offering solutions. These techniques mirror those that we expected the advisors to use with their clients.

Phase Two: Pilot testing of intervention acceptability and fidelity

Piloting delivery of the initial intervention and assessment of fidelity was carried out at the same time as piloting the study procedures necessary for the cluster randomised trial that will be used to evaluate the final version of the intervention. The process of intervention development was iterative so that the theoretical modelling and the results from piloting the intervention informed further data collection and analysis.

Methods

Study design

Participants

Any stop smoking advisors working in the London boroughs of Tower Hamlets or City and Hackney, and in a community pharmacy allocated to the intervention arm of the pilot trial, were eligible to participate in the STOP training. All advisors had completed NCSCT level two training (details of the pilot trial have been published previously[30]). There were no restrictions on time elapsed since

For peer review only - http://bmjopen.bmj.com/site/about/guidelines.xhtml

training as a stop smoking advisor. Advisors could be either pharmacists or other pharmacy workers such as counter assistants, providing they were trained to deliver stop smoking services.

Acceptability of intervention

i) Attendance.

Details on attendance at each training session and any reasons given for non-attendance were documented.

ii) Qualitative assessment.

All participants who consented to participate in the trial, regardless of whether they attended any training sessions, were invited to attend a semi-structured interview one month after training. Views about the intervention and practical implementation were elicited. We also explored barriers and facilitators to attending training sessions and implementing the intervention. The inclusion of individuals who did not attend training allowed their perspectives on the barriers to attendance to be considered. Interviews were conducted by a researcher independent of intervention delivery (RS) to minimize risk of social desirability responses. A framework analysis of the data[31] was conducted by RS.

Intervention fidelity and implementation in practice

We assessed fidelity of the intervention in terms of improving smoker engagement by using simulated clients, a method previously used for similar assessments in community pharmacy.[32] Two actors visited each pharmacy on different days, without prior knowledge of the pharmacy, although this had been agreed to at initial study consent. The actors presented different clinical scenarios designed to give an opportunity for counter staff to engage them with the smoking cessation service. A matrix was used to ensure that two different case scenarios were presented to each pharmacy. Both actors were familiar with portraying simulated patients in clinical scenarios in Objective Structured Clinical Examinations (OSCE) for medical students.

Each actor completed a checklist following the visit which included items related to their interaction with the advisor e.g. whether a client would like to attend the smoking cessation service, general advisor communication style and use of study materials such as tar jars, stop smoking posters etc. Both actors underwent training before visiting pharmacies and were blind to training status.

Self-Efficacy

For peer review only - http://bmjopen.bmj.com/site/about/guidelines.xhtml

An important hypothesized theoretical mechanism for change in advisor behaviour was through increased self-efficacy. We assessed this using a validated 0 - 5 point Likert scale, previously used in a similar context[33] such that higher ratings indicated greater self-efficacy. The scale was sent for self-completion one month after training to all advisors who attended.

RESULTS

Intervention Acceptability

All twelve stop smoking advisors in the intervention pharmacies agreed to participate in the training programme and gave informed consent. In total six advisors completed both training sessions (three at the group programme and three at a separate in-house training session). One participant withdrew after session one due to sickness and another because of low perceived relevance of the training sessions. Four participants did not attend any training for reasons detailed below.

Eight of the twelve advisors agreed to interview, four of whom had attended both training sessions, one had attended only session one and three had not attended any training. Six of those interviewed were pharmacists, one was a counter assistant and one a pharmacy technician (age range 24-52 years). Five interviewees were male. Themes emerging from the analysis of interview transcripts that were related to the training are presented in Table 1.

Table 1. Summary of qualitative findings from interviews with smoking cessation advisors related to intervention training

Theme	Illustrative Quotation
**Logistics/organisation of training**	*I think it was way out …I've never been there before, and because I close six thirty, …And I can't just leave at six thirty on the dot, I've got to tidy up things. So sometimes I don't leave here until maybe seven twenty or seven thirty,*
**Suggestions to improve the logistics of training**	*For us as pharmacists it's difficult because we need to have ourselves covered by another pharmacist, otherwise the business can't run. So unless there's compensation for getting a locum pharmacists*
**Training structure, content including the social media support element**	*Yeah it was great…got us involved…It was very informative, the way they actually made us do some play-acting to actually show a point to why they were doing that*

For peer review only - http://bmjopen.bmj.com/site/about/guidelines.xhtml

	particular part of the talk. So that was all good.<br><br>*to join (facebook) you would have to enrol as a company. So I don't think the boss really wanted Facebook as a company,... (Pharmacist)*
**Reflection on skills learnt**	*So I think this research should carry on for another year, not just six months. Because obviously we as practitioners need time...Because it's something new to us as well...There's more learning for us to do. It takes time for us to participate and engage.*<br>*Like when using the scales, when I try and engage customers I'll ask them on a scale of one to ten how ready are you to give up, one being not so ready and ten being very ready. So using those trigger questions they're very good, in that little book that we were given.*
**Improvement suggestions for training structure and content**	*I wonder whether there's an ability for the (other pharmacy) staff to be let off work maybe at a training session just for staff...or it could be a session where someone from your they can maybe..., come in for half an hour and do a similar thing to what we did. But not that in-depth that we were trained.*
**Application of learnt skills in practice and outcomes**	*When you come up to someone, say they came in and then they don't come back for a few weeks and then come back again, it's actually to keep the people in. So all the bits we've learnt additionally that we found now we've got a better success rate because people are coming back. Because we're saying...like sometimes if you talk to someone and you say oh well, you've smoked today, you shouldn't have really done that, but now we don't say that. We say look, this is a way to try around that, let's see what else we can try to stop you getting into that situation. That sort of thing. So we're using different ideas with them*

Logistics were identified as an important barrier to effective use of the training sessions, with participants reporting difficulty with both location (at a central training site) and timing (in the evenings 7pm-9.30pm). This was despite exploratory work that suggested evenings would be more convenient. Recommendations for venues closer to pharmacies and potential reimbursement to enable locum pharmacists to cover for pharmacist absence were made. The overall structure and content of the face-to-face training was acceptable and participants engaged well with activities, including role-play.

Only one advisor registered for the Facebook element of the intervention. Reasons for not engaging included not being allowed to use social media by managers, feeling uncomfortable with use of

For peer review only - http://bmjopen.bmj.com/site/about/guidelines.xhtml

social media, and concern related to combining social and working lives. Participants reported that they felt able to use the newly learnt skills in practice and reported using specific techniques, for example confidence scales, to assist in consultations. Some participants, however, felt that it would take time for the new skills to become fully embedded in routine practice.

Fidelity assessment

Actor one visited all twelve intervention pharmacies, but actor two visited only nine pharmacies, because of limited availability. Table 2 shows ratings for the 18 items of the checklist. Actor two appeared to rate pharmacies more negatively than actor one and there was some inconsistency between responses for what might be expected to be objective items, for example the presence of a stop smoking poster and smoking prompts such as presence of a tar jar. Few pharmacy workers with whom the actors came into contact had attended the training (approximately 10%) as they were counter assistants not specifically trained in smoking cessation.

Table 2. Actor ratings for assessment of fidelity including display of smoking cessation materials, advisor communication skills and content of the consultation

	Actor 1		Actor 2	
	Yes	No	Yes	No
NHS Stop Smoking Service poster displayed	7	5	9	0
Audio-visual information about the NHS Stop Smoking Service	0	12	0	9
Leaflets about the NHS Stop Smoking Service	9	3	6	3
Smoking prompts e.g. tar jar	4	8	1	8
Were other clients observed being asked about smoking?	0	12	0	9
Good body language	6	6	6	3
Good listening skills	3	9	6	3
Use open questions	2	10	3	6
Was topic of smoking raised	0	12	0	9

For peer review only - http://bmjopen.bmj.com/site/about/guidelines.xhtml

Was smoking raised directly	1	11	0	9
Was smoking raised indirectly	0	12	0	9
Was client told there is a smoking cessation service	6	6	0	9
Was service highlighted as free aside from NRT	4	8	0	9
Was it service highlighted as delivered by experts	0	12	0	9
Was it highlighted 4x higher success rate with programme	2	10	0	9
Asked whether client want referral to the service	4	8	0	9
Assess whether client ready to change	2	10	0	9
Close by saying door is always open	5	7	0	9

Self-Efficacy Ratings

All 11 advisors completed the pre-training questionnaire and 10 completed questionnaires post-training. For pre-training, median score was 4 (range 3.5 to 4.6) and post training 4.5 (4.0-5.0).

Phase Three: Intervention Refinement

Key issues arising from the pilot and fidelity assessments that needed to be addressed in revising the STOP intervention were:-

- Ensuring that all pharmacy workers in contact with smokers were trained in smoker engagement (e.g. all counter assistants and not just those trained as smoking cessation advisors).
- Encouraging full attendance at the training sessions and display of study materials, financial reward (£30 cash per participant per session).
- Understanding organisational barriers to implementation of the intervention in community pharmacies and how these might be overcome.

In response to these issues we used realist review techniques to synthesise previous research on implementation of complex interventions for smoking cessation in community pharmacies, aiming to identify factors associated with success or failure of the intervention. The analysis took into account the environment in which the intervention was delivered and factors associated with the recipient,

For peer review only - http://bmjopen.bmj.com/site/about/guidelines.xhtml

together with features of the intervention itself that might influence effectiveness.[34] We gained insight into how specific interventions achieved their outcomes and, where there was an intervention that was only partially effective, the review indicated areas where aims had been achieved and the reasons why they might not have been achieved. The preliminary realist analysis identified four mechanisms upon which previously reported interventions had been operating: pharmacist identity; pharmacist capability; pharmacist motivation; stakeholder confidence. These mechanisms highlight specific attributes both of pharmacists and the wider organisational context that could potentially be exploited to enhance the effectiveness of pharmacy interventions for smoking cessation (Supplementary Table 1).[35]

We selected Diffusion of Innovations Theory (as adapted by Greenhalgh et al. for the study of organisational innovation in health care[36]) to seat the intervention within the organisational context of community pharmacies. This theory can be used to address how new methods of service delivery can be embedded and sustained in organisations. Amendments to the intervention according to the results of the initial piloting and review of theory are illustrated in Box 1 in italics. An overall description using the TIDieR framework[37] – a tool to facilitate explicit reporting of interventions – is presented in Supplementary Table 2.

The programme theory giving an overview of the intended operation of the intervention represented as a logic model[38] is shown in Figure 2.

INSERT FIGURE 2 ABOUT HERE

DISCUSSION

Statement of principal findings

We developed the STOP intervention in line with current Medical Research Council guidance for complex interventions, incorporating information from previous literature and results of our qualitative work with cessation advisors. We drew on theories about psychological aspects of human behaviour to develop our initial intervention. Interviews with pharmacy workers after the pilot study suggested that the face-to-face training was well received by those attending and that such training might improve self-efficacy in delivering smoking cessation interventions. However only six of 12

For peer review only - http://bmjopen.bmj.com/site/about/guidelines.xhtml

participants attended both training sessions and four individuals did not attend any sessions, suggesting a need for changing recruitment processes.

Our realist review[35] suggests that interventions to bring about behaviour change in pharmacy workers need to take into account role-identity, capability, motivation and the wider organisational context in which the intervention is operating. Behaviour change requires practitioner practice and continuing support with feedback[39] both to develop skills and to overcome anxieties about implementing a new form of practice. In the initial intervention this support was anticipated to be available through social media, but given that this was not readily accepted, a more traditional approach of follow-up sessions within practice is proposed for the revised intervention.

Social media is a relatively new phenomenon and currently seen as an area of great potential for many behavioural interventions,[40] however, our study identifies possible operational barriers that need to be considered. For example, health care professionals may not find it acceptable to blur the boundaries between professional and social life. For this form of intervention to be successful it may be necessary to give greater consideration to the motives and barriers towards using social media.[41] We selected 'Facebook' as one of the most well known forms of social media and hence one we anticipated participants to be familiar with, however it is possible that different social networking forums may have been more acceptable.

Explicit attention needs to be given to the implementation of the intervention and integration with routine practice. Previous systematic reviews in other fields have similarly highlighted the importance of implementation.[42] In the revised STOP intervention we addressed effective implementation using Diffusion of Innovations theory. This theory aims to explain how new ideas spread in an organisational system and was useful in identifying key items that we needed to include in the intervention; for example allaying concerns before adoption and demonstrating that the intervention is not complex and fits well with the expanded role of community pharmacies. We also highlighted the need for flexibility in how the training sessions are delivered, for example at a central location or in individual pharmacies. The revised intervention training will be offered at different times of day and on different days of the week and those who are unable to attend will be offered on-site training.

For peer review only - http://bmjopen.bmj.com/site/about/guidelines.xhtml

Comparison with other studies

The pilot findings from STOP are in line with previous studies that found smoking cessation training acceptable to pharmacy workers.[9 10] The finding that logistics and organizational issues may act as a barrier to implementation is also in line with evidence that potentially effective interventions for smoking cessation advisors have been developed but may not have changed quit rates or uptake of smoking cessation services.[43] The explicit application of organisational theory in development of the STOP intervention is an attempt to address this problem.

A realist review by our own team, undertaken in parallel with the fieldwork on this complex intervention and feeding into its final refinement, confirmed two key aspects of our empirical findings. Firstly, that training in both factual knowledge and skills to enhance self-efficacy, informed by theories of behaviour change, improves the capacity and confidence (capability and motivation) of front-line pharmacy staff in supporting smoking cessation. Secondly, that organisational barriers (opportunity) may be significant even when individual motivation and capability are targeted. An additional finding from the realist analysis of the literature suggests that uptake and sustainability of pharmacy-based smoking cessation support depends on public and professional trust in the extended role of the pharmacist, which in turn depends on positive messages from the media and professional bodies. In the revised intervention we recommend support from local champions such as Healthy Living Leads, which may partly address this issue.

Strengths and weaknesses

A strength of the current study was the development work prior to piloting the intervention and using a recommended behavioural intervention development system to guide this.[19] We conducted qualitative work in community pharmacies, which helped us to gain an understanding of the detailed interactions between cessation advisors and their clients and used this to develop and refine the intervention. We concurrently developed a theoretical framework to underpin the intervention, drawing both from psychological theories related to behaviour change and organisational theory attempting to explain how interventions become incorporated into routine practice. Having established a framework, we translated this into behaviour change techniques to facilitate change in our three target behaviours: i) increased engagement of smokers; ii) retaining individuals in the stop smoking service; iii) improving quit rates. Our work builds upon the behaviour change techniques thought to be most effective for smoking cessation[16] but which may not be well implemented in routine practice.[43]

For peer review only - http://bmjopen.bmj.com/site/about/guidelines.xhtml

It is a further strength of the current study that we addressed organisational aspects likely to influence the effectiveness of the interventions. Studies developing smoking cessation interventions to date have mainly focussed on theories of individual behaviour change such as the transtheoretical model.[44] Whilst studies designed to test this approach show some benefit,[45] such theories tend not to take into account broader considerations of how the intervention might be implemented. We are not aware of previous programmes in community pharmacies that have explicitly used organisational theory in addition to psychological theory to develop an intervention. Although some previous studies have compared different models of service delivery, these have not provided a theory-based exploration of the mechanisms by which the intervention brought about behaviour change in different settings.[46 47] As a result it is difficult to generalise from these trials because mechanisms may be operating differently in different community pharmacies.[48] A consideration of theoretical perspectives on the implementation of interventions to supplement psychological theories of behaviour change would seem prerequisite if the intervention is to work reliably in diverse settings.

Our assessment of Intervention fidelity is also a strength.[43] In this study we used simulated clients to assess the degree to which core elements of the intervention were reflected in engagement behaviour of pharmacy staff. These methods have previously been used successfully in community pharmacies.[32 49 50] The simulated client methodology was informative in identifying areas where the intervention needed increased focus, for example the need to extend training to all community pharmacy workers. Whilst the simulated client visits provided a qualitative assessment of intervention fidelity and engagement behaviour, it was a weakness that there was no quantitative assessment, due to the small scale of the pilot study. In addition we found some discrepancy between actors in scoring apparently objective measures such as presence or absence of stop smoking posters. This raises questions over the reliability of this method and the need for increased training of actors to ensure any assessment is robust.

A weakness of the study was that both our pre-intervention development (qualitative studies) and piloting drew upon the experience of pharmacies which were sufficiently engaged in the topic area to participate in a research study. There may be additional barriers to implementation for pharmacies that do not see smoking cessation as a priority. In addition there may be practical problems such as lack of a private consulting room, which could make implementation difficult in some pharmacies. The recent development of the Healthy Living Pharmacy framework[2] will mean

For peer review only - http://bmjopen.bmj.com/site/about/guidelines.xhtml

that pharmacies are increasingly better equipped for smoking cessation and other health behaviour change tasks. It is therefore timely for pharmacy workers to be suitably trained in behaviour change skills.

A limitation of the qualitative data from the advisor interviews was that advisors' self-report of their experience of attending training sessions may be subject to social desirability responses. Nevertheless, the fact that a researcher independent from the intervention team conducted the interviews adds some validity to this assessment.

Implications for clinical practice and policy makers

We found that smoking cessation advisors were generally motivated to support their clients to change health behaviours and were receptive to additional skills training. However we identified a gap in referral of smokers from non smoking cessation trained counter assistants to trained smoking cessation advisors. This suggests that in order to engage smokers more effectively, a wider work force than stop smoking advisors needs to be trained. Nevertheless the results from our pilot studies provide initial evidence that STOP may be an acceptable and potentially effective intervention.[30]

Implications for future research

Whilst many health systems are expanding the role of community pharmacies into lifestyle management, there has been very little research on the effectiveness of pharmacy workers in their extended roles.[51] Since there are substantial economic implications for funding wider delivery of primary health care through pharmacies, health commissioners may need stronger evidence before decisions on allocation of funding are made. Such evidence will require large-scale studies to evaluate the effectiveness and cost effectiveness of changes in service delivery and further investigation into the organisational barriers to implementation.

Additional Information

The training and intervention materials, including the intervention manual, will be available upon request from the authors following completion of the evaluation phase of the STOP programme.

Acknowledgments The authors thank participants and their community pharmacies. This paper presents independent research funded by the National Institute for Health Research (NIHR) under its

For peer review only - http://bmjopen.bmj.com/site/about/guidelines.xhtml

Programme Grants of Applied Research Programme. The views expressed are those of the authors and not necessarily those of the NHS, the NIHR or the Department of Health. We thank the independent members of the expert advisory group Professors Hayden MacRobbie and Rob Horne.

Contributors

This study is a sub-study of the NIHR funded STOP programme on which RW is chief investigator and CG, SE, ST and TG are co-investigators. LS led development of the initial version of the intervention and designed the fidelity assessment. RS, W-Y J, SJ, AC, CR, VM and EE recruited sites and collected and analysed qualitative and quantitative data. FM and TG conducted the realist analysis. AT and TG gave critical comments on the manuscript. LS and RW drafted the paper with input from all other authors. SJ assisted in refining the intervention. All authors have seen and approved the final manuscript.

Competing interests None.

Funding: NIHR Programme grant RP-PG-0609-10181

Participant consent Written informed consent obtained.

Ethics approval Ethical approval for the study was obtained from the NRES Committee South Central - Berkshire B (reference number: 13/SC/0189).

Provenance and peer review Not commissioned; externally peer reviewed

Data sharing statement

No additional data available.

Open Access This is an Open Access article distributed in accordance with the terms of the Creative Commons Attribution (CC BY 4.0) license, which permits others to distribute, remix, adapt and build upon this work, for commercial use, provided the original work is properly cited. See: http://creativecommons.org/licenses/by/4.0/

For peer review only - http://bmjopen.bmj.com/site/about/guidelines.xhtml

Figure legends

Figure 1. The Double Whammy paper based desk prompt

The desk prompt presents key messages from the training in a calendar format, displaying examples of how issues may be raised and addressed.

Figure 2. STOP programme theory

Logic model of the intended operation of the intervention.

For peer review only

For peer review only - http://bmjopen.bmj.com/site/about/guidelines.xhtml

References

1. Anonymous. Pharmacy in England. Building on strengths – delivering the future. In: Health, ed. London: H M Government, 2008.
2. Donovan GR, Paudyal V. England's Healthy Living Pharmacy (HLP) initiative: Facilitating the engagement of pharmacy support staff in public health. *Res Social Adm Pharm* 2016;12(2):281-92. doi: 10.1016/j.sapharm.2015.05.010
3. PSNC. Community Pharmacy Contractual Framework: Pharmaceutical Services Negotiating Committee; 2016 [Available from: http://psnc.org.uk/contract-it/the-pharmacy-contract/.
4. Lifestyle statistics Health and Social Care Information Centre. Statistics on NHS Stop Smoking Services: England, April 2012 – March 2013, 2013.
5. NHS Employers. Pharmacy-based stop smoking services: optimising commissioning. London: NHS Employers, 2009.
6. Lindsey L, Husband A, Steed L, et al. Helpful advice and hidden expertize: pharmacy users' experiences of community pharmacy accessibility. *J Public Health (Oxf)* 2016 doi: 10.1093/pubmed/fdw089
7. Eades C, Ferguson J, O'Carroll R. Public health in community pharmacy: A systematic review of pharmacist and consumer views. *BMC Public Health* 2011;11(1):582.
8. Jubraj B, Barnett NL, Grimes L, et al. Why we should understand the patient experience: clinical empathy and medicines optimisation. *Int J Pharm Pract* 2016;24(5):367-70. doi: 10.1111/ijpp.12268
9. Sinclair H, Bond C, Lennox, AS, et al. An evaluation of a training workshop for pharmacists based on the Stages of Change model of smoking cessation. *Health Education Journal* 1997;56:296-312.
10. Sinclair HK, Bond CM, Lennox AS, et al. Training pharmacists and pharmacy assistants in the stage-of-change model of smoking cessation: a randomised controlled trial in Scotland. *Tob Control* 1998;7(3):253-61.
11. Bock BC, Hudmon KS, Christian J, et al. A tailored intervention to support pharmacy-based counseling for smoking cessation. *Nicotine Tob Res* 2010;12(3):217-25. doi: 10.1093/ntr/ntp197
12. Maguire TA, McElnay JC, Drummond A. A randomized controlled trial of a smoking cessation intervention based in community pharmacies. *Addiction* 2001;96(2):325-31. doi: 10.1080/09652140020021062
13. Sinclair HK, Silcock J, Bond, CM, et al. The cost-effectiveness of intensive pharmaceutical intervention in assisting people to stop smoking. *The International Journal Of Pharmacy Practice* 1999;7:107-12.
14. Sinclair HK, Bond CM, Lennox AS. The long-term learning effect of training in stage of change for smoking cessation: a three-year follow up of community pharmacy staff's knowledge and attitudes. *International Journal of Pharmacy Practice* 1999;7(1):1-11.
15. Sinclair HK, Bond CM, Stead, LF et al. Community pharmacy personnel interventions for smoking cessation. *Cochrane Database of Systematic Reviews Issue 1 Art No: CD003698 DOI: 101002/14651858CD003698pub2* 2008:1-15.
16. West R, Walia A, Hyder N, et al. Behavior change techniques used by the English Stop Smoking Services and their associations with short-term quit outcomes. *Nicotine Tob Res* 2010;12(7):742-7. doi: 10.1093/ntr/ntq074
17. MRC. A framework for devleopment and evaluation of RCTs for complex interventions to improve health 2000 [Available from: https://www.mrc.ac.uk/documents/pdf/rcts-for-complex-interventions-to-improve-health/.
18. MRC. Developing and evaluating complex interventions: new guidance. 2008;https://www.mrc.ac.uk/documents/pdf/complex-interventions-guidance/

For peer review only - http://bmjopen.bmj.com/site/about/guidelines.xhtml

19. Michie S, Atkins L, West R. The Behaviour Change Wheel. A Guide to Designing Interventions: Silverback Publishing 2014.
20. Michie S, West R. The behaviour change wheel: A new method for characterising and designing behaviour change interventions. *Implementation Science* 2011;6
21. Steed L, Kassavou A, Madurasinghe VW, et al. Community pharmacy interventions for health promotion: effects on professional practice and health outcomes. *The Cochrane Library* 2014
22. Brown TJ, Todd A, O'Malley C, et al. Community pharmacy-delivered interventions for public health priorities: a systematic review of interventions for alcohol reduction, smoking cessation and weight management, including meta-analysis for smoking cessation. *BMJ Open* 2016;6(2):e009828. doi: 10.1136/bmjopen-2015-009828
23. Carson KV, Verbiest ME, Crone MR, et al. Training health professionals in smoking cessation. *Cochrane Database Syst Rev* 2012(5):CD000214. doi: 10.1002/14651858.CD000214.pub2
24. Sohanpal R, Rivas C, Steed L, et al. Understanding recruitment and retention in the NHS community pharmacy stop smoking service: perceptions of smoking cessation advisers. *BMJ Open* 2016;6(7):e010921. doi: 10.1136/bmjopen-2015-010921
25. Habermas J. The Theory of communicative action, reason and the rationalisation of society. : Heineman 1984.
26. Bandura A. Self-efficacy: Toward a unifying theory of behavioral change. *Psychological Review* 1977;84(2):191-215.
27. Deci EL, Ryan RM. Intrinsic motivation and self-determination in human behaviour. New York: Plenum 1985.
28. RCGP. Inquiry into Patient Centred Care in the 21st Century 2014 [14.8.16]. Available from: http://www.rcgp.org.uk/policy/rcgp-policy-areas/inquiry-into-patient-centred-care-in-the-21st-century.aspx.
29. Michie, S., Richardson, M., Johnston, M., Abraham, C., Francis, J., Hardeman, W., Wood, C. E. (2013). The behavior change technique taxonomy (v1) of 93 hierarchically clustered techniques: building an international consensus for the reporting of behavior change interventions. *Ann Behav Med, 46*(1), 81-95.
30. Madurasinghe VW, Sohanpal R, James W, et al. Smoking treatment optimisation in pharmacies (STOP): a cluster randomised pilot trial of a training intervention. *Pilot and Feasibility Studies*;2017; 3:1 doi: 10.1186/s40814-016-0120-9
31. Richie, J and Spencer, L (1994), 'Qualitative data analysis for applied policy research', in Bryman and Burgess, eds., Analysing Qualitative Data, London: Routledge, p173-194.
32. Watson MC, Skelton JR, Bond CM, et al. Simulated patients in the community pharmacy setting. Using simulated patients to measure practice in the community pharmacy setting. *Pharm World Sci* 2004;26(1):32-7.
33. Brose LS, West R, Michie S, et al. Evaluation of Face-to-Face Courses in Behavioural Support for Stop Smoking Practitioners. *Journal of Smoking Cessation* 2012;7:25-30. doi: doi:10.1017/jsc.2012.6
34. Pawson R, Greenhalgh T, Harvey G, et al. Realist review--a new method of systematic review designed for complex policy interventions. *Journal of health services research & policy* 2005;10 Suppl 1:21-34. doi: 10.1258/1355819054308530 [published Online First: 2005/08/02]
35 .Greenhalgh T, McFarlane F, Steed L et al. What works for whom in pharmacist led smoking cessation services: A realist review BMC Medicine (2016) 14:209

For peer review only - http://bmjopen.bmj.com/site/about/guidelines.xhtml

36. Greenhalgh T, Robert G, Macfarlane F, et al. Diffusion of Innovations in Service Organizations: Systematic Review and Recommendations. *Milbank Quarterly* 2004;82(4):581-629. doi: 10.1111/j.0887-378X.2004.00325.x
37. Hoffmann TC, Glasziou PP, Boutron I, et al. Better reporting of interventions: template for intervention description and replication (TIDieR) checklist and guide. *BMJ* 2014;348 doi: 10.1136/bmj.g1687
38. FOUNDATION WKK. Using Logic Models to Bring Together Planning, Evaluation, and Action Logic Model Development Guide. One Michigan Avenue, East, Battle Creek, MI 49017-4012 USA. http://www.wkkf.org2006.

39. National Institute for Clinical Excellence Behaviour Change Individual Approaches. PH49. 2014 https://www.nice.org.uk/guidance/ph49/resources/behaviour-change-individual-approaches-1996366337989

40. Pagoto S, Waring ME, May CN, et al. Adapting Behavioral Interventions for Social Media Delivery. *J Med Internet Res* 2016;18(1):e24. doi: 10.2196/jmir.5086
41. Antheunis ML, Tates K, Nieboer TE. Patients' and health professionals' use of social media in health care: motives, barriers and expectations. Patient Educ Couns. 2013 Sep;92(3):426-31. doi: 10.1016/j.pec.2013.06.020.
42. Taylor S, Pinnock H, Epiphanou E, et al. A rapid synthesis of the evidence on interventions supporting self-management for people with long-term conditions: PRISMS. *Health Serv Deliv Res* 2014;2(53)
43. Lorencatto F, West R, Christopherson C, et al. Assessing fidelity of delivery of smoking cessation behavioural support in practice. *Implement Sci* 2013;8:40. doi: 10.1186/1748-5908-8-40
44. Prochaska JO, DiClemente CC. Stages and processes of self-change of smoking: toward an integrative model of change. *J Consult Clin Psychol* 1983;51(3):390-5.
45. Armitage CJ, Arden, M.A.,. How Useful Are the Stages of Change for Targeting Interventions? Randomized Test of a Brief Intervention to Reduce Smoking. *??* 2008;27(6):789-98.
46. Bauld L, Chesterman J, Ferguson J, et al. A comparison of the effectiveness of group-based and pharmacy-led smoking cessation treatment in Glasgow. *Addiction* 2009;104(2):308-16. doi: 10.1111/j.1360-0443.2008.02446.x
47. McEwen A, West R, McRobbie H. Effectiveness of specialist group treatment for smoking cessation vs. one-to-one treatment in primary care. *Addict Behav* 2006;31(9):1650-60. doi: 10.1016/j.addbeh.2005.12.014
48. Nkansah N, Mostovetsky O, Yu C, et al. Effect of outpatient pharmacists' non-dispensing roles on patient outcomes and prescribing patterns. *Cochrane Database Syst Rev* 2010(7):CD000336. doi: 10.1002/14651858.CD000336.pub2
49. Watson MC, Cleland JA, Bond CM. Simulated patient visits with immediate feedback to improve the supply of over-the-counter medicines: a feasibility study. *Fam Pract* 2009;26(6):532-42. doi: 10.1093/fampra/cmp061
50. Inch J, Porteous T, Maskrey V, et al. It's not what you do it's the way that it's measured: quality assessment of minor ailment management in community pharmacies. *Int J Pharm Pract* 2016 doi: 10.1111/ijpp.12305
51. Mossialos E, Naci H, Courtin E. Expanding the role of community pharmacists: policymaking in the absence of policy-relevant evidence? *International (Sweden / Canada / Belgium)* 2013;111(2):135-48. doi: 10.1016/j.healthpol.2013.04.003

For peer review only - http://bmjopen.bmj.com/site/about/guidelines.xhtml

30

For peer review only

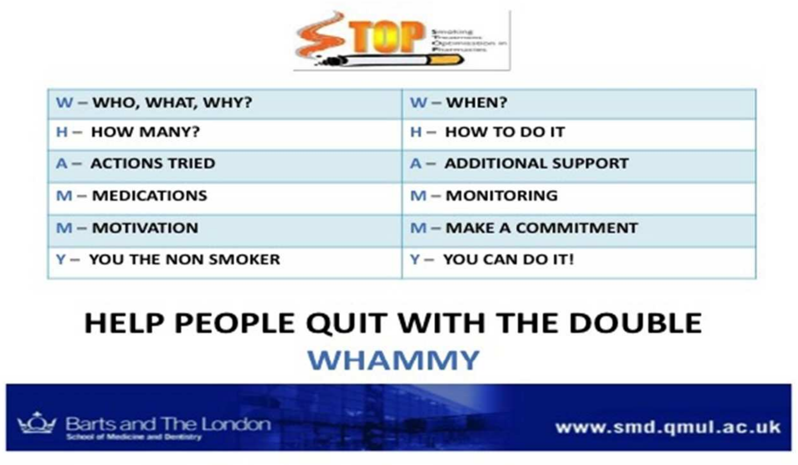

Figure 1. The Double Whammy paper based desk prompt

254x190mm (72 x 72 DPI)

For peer review only - http://bmjopen.bmj.com/site/about/guidelines.xhtml

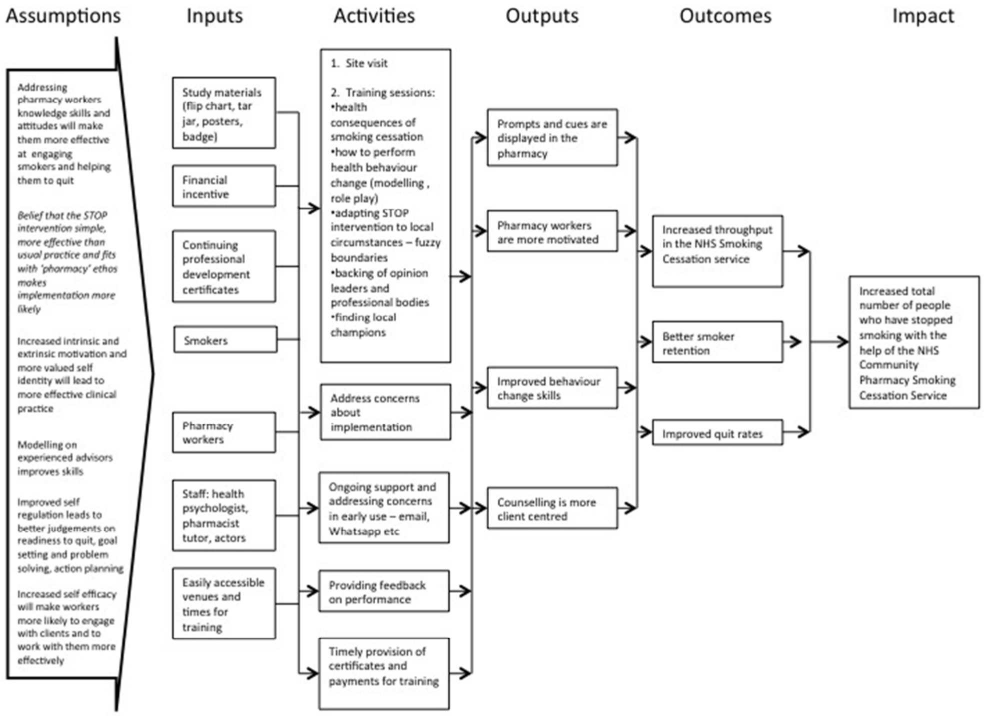

Figure 2. STOP programme theory

254x190mm (72 x 72 DPI)

Supplementary Table 1. Summary of preliminary findings from realist review

Mechanism by which the pharmacy smoking cessation service might be promoted	How the mechanism might be strengthened	Contextual influences
**Pharmacist identity**	• Strengthen 'pharmacy' identity by emphasising backing from professional bodies<br>• Promote non-medication and public health roles of the pharmacist<br>• Encourage patient-orientation rather than product-orientation<br>• Encourage a professional as opposed to 'technical' ethos	• Undergraduate education promotes these characteristics<br>• Professional bodies embrace extended role<br>• Policymakers recognise pharmacists as professionals
**Pharmacist capability**	• Strengthen knowledge base on health behavior change<br>• Consultation skills training<br>• Easily accessible educational sessions<br>• Change beliefs and attitudes, boosting self-efficacy in delivering the smoking cessation and encouraging belief that the intervention will be effective	• Quality, depth and breadth of training<br>• Training addresses skills and attitudes as well as knowledge<br>• Accessibility of training throughout professional life
**Pharmacist motivation**	• Present business arguments eg diversification of revenue streams, investment in space for financial returns<br>• Recognise training as continuing professional development	• Involvement of other pharmacies and pharmacists in health behavior change establishing a professional norm.<br>• Strong business model justifying investment in infrastructure<br>• Simple system for claiming payments
**Stakeholder confidence**	• Build confidence in the intervention from government, professional bodies (general practitioner and pharmacy), health commissioners.<br>• Change perceptions of patients and carers about the position of the pharmacist in health care system.	• Clear, positive messages in the media about the extended role<br>• Positive reaction to the role from other branches of primary care at national and local level

1

For peer review only

Supplementary Table 2. The intervention to be evaluated in the STOP randomised controlled trial described using the Template for Intervention Description and Replication (TIDieR) checklist.*

**Item 1. Brief name: Provide the name or a phrase that describes the intervention**	STOP - Service optimisation and communication skills training to strengthen the NHS smoking cessation service in community pharmacies.
**Item 2. Why: Describe any rationale, theory, or goal of the elements essential to the intervention**	We used the COM-B as a starting framework recognising the three behaviours of i) engaging smokers, ii) retaining smokers in the service and iii) smokers quit rates. For each we applied further theories including Social Cognitive theory [26] to underpin development of the intervention. The intervention aims to boost self-efficacy and self-regulation in the pharmacy staff to improve skills promoting recruitment and retention of service users and increasing quit success. We also drew upon Self Determination theory[27] to influence motivation which we see as important for the pharmacy staff to react to opportunities to promote the service and to create opportunities to discuss the service with potential clients.<br><br>We mapped the theoretical constructs onto specific behaviour change techniques using the behaviour change wheel and COM-B behaviour change model.[19 20] This model is increasingly used in development of behavioural interventions and has previously been used for smoking cessation. These behaviour change techniques were then embedded within the intervention.<br><br>*For implementation of the intervention we applied Diffusion of Innovations theory [34] which enabled us to seat the intervention in the complex organisational structures which we found both within and between community pharmacies.*<br><br>*The training stresses the advantages over usual practice in terms of revenue for the pharmacy and in building up the common good. The intervention is brief and fits in with usual practice and work flows and can be adapted in the context of each individual pharmacy. Concerns will be addressed about risks to profits – more important for the pharmacist/owners than for the staff. Backing from local opinion leaders will be emphasised (Hemant Patel, Secretary of the Local Pharmaceutical Committee) and we suggest that individual champions are appointed within each pharmacy perhaps also with a monthly 'STOP smoking day' to*

For peer review only - http://bmjopen.bmj.com/site/about/guidelines.xhtml

	maintain the focus of attention.
**Item 3. What (materials): Describe any physical or informational materials used in the intervention, including those provided to participants or used in intervention delivery or in training of intervention providers. Provide information on where the materials can be accessed**	Training material will be given as printed handouts in a study folder. The training sessions will involve role-plays and training videos – targeting engagement of smokers and optimising delivery of the stop smoking service aiming to increase retention and successful quitting. Attendees will be given a STOP flip chart (for desk) – with notes on key behaviour change techniques, 'Quit card' (can be kept in wallet) – to be given to smoker clients to reinforce smoking cessation strategies.<br>*The attendees will be given badges to wear in pharmacy as visual prompts to potential service users to ask about smoking cessation.*<br>*Social media will be used to perform a 'helpdesk' function using email and 'Whatsapp' to provide an easily accessible 'one-stop' platform to access study materials such as training videos. We will also signpost external smoking cessation resources using Whatsapp for example smoking cessation training websites, and latest cessation guidance.*<br>*All materials will be made available on a website to facilitate access by those not using social media.*
**Item 4. What (procedures): Describe each of the procedures, activities, and/or processes used in the intervention, including any enabling or support activities**	The training sessions will be face to face in a group of approximately 12 stop smoking advisors or pharmacy support staff. The STOP training will be incentivised for pharmacy staff by providing a cash amount of £30 to attend each session including travel expensed (£60 for both sessions) and a 'certificate of completion' to each staff member for attending and completing the training (see Appendix 2 and 3). The payment will be made only on completing the second session.
**Item 5. Who provided: For each category of intervention provider (for example, psychologist, nursing assistant), describe their expertise, background and any specific training given**	Training sessions will be facilitated by an experienced trainer/health psychologist and a community pharmacist, also a trainer. Skilled facilitation is needed for these groups, supported by the leader/co-leader structure and the pharmacists are then also available to answer practical questions about smoking cessation which we found arose frequently in the sessions that we conducted for the pilot study.
**Item 6. How: Describe the modes of delivery (such as face to face or by some other mechanism, such as**	The face-to-face training will be delivered at varying times to accommodate the work routine of pharmacy staff.<br>See also Item 3 above for internet and social media resources.

For peer review only

**internet or telephone) of the intervention and whether it was provided individually or in a group**	
**Item 7. Where: Describe the type(s) of location(s) where the intervention occurred, including any necessary infrastructure or relevant features**	The training will be delivered in training venues commonly used by pharmacy staff. Example of venues include the Tomlinson Centre in City and Hackney, Newham hospital or restaurants with separate meeting room in Newham. Venues will be chosen to be easily accessible to pharmacy workers after the pharmacy closes in the evening. Suggestions for venues will be taken from local Public Health commissioners and from the pharmacists themselves at recruitment.<br><br>Training sessions for pharmacy workers for other purposes are often held at restaurants where a meal or vouchers are provided and we will fit in with this model if possible if an appropriate meeting room is also available.
**Item 8. When and how much: Describe the number of times the intervention was delivered and over what period of time including the number of sessions, their schedule, and their duration, intensity or dose**	The training will comprise two sessions, each session of 2.5 hours, separated by two weeks. Pilot work suggested that longer sessions would not be more effective.<br><br>Tasks will be given at the end of the first session for advisors to complete NCSCT training (level 1 or 2) and to consider how the intervention could be adapted to work in their context.<br><br>Other elements of the intervention such as badges for workers and the flip chart will act as visual cues to remind workers of the training. *Badges will be worn during the working day and the flip charts will be kept in the pharmacy consulting room.*
**Item 9. Tailoring: If the intervention was planned to be personalised, titrated or adapted, then describe what, why, when, and how**	The content of the training will be adjusted according to advisor/pharmacy staff status. Pharmacy staff will attend only the first training session where engagement of smokers is discussed and advisors will attend both sessions.
**Item 10. Modifications: If the intervention was modified during the course of the study,**	*The intervention comprises an irreducible core of communication and behaviour change skills training, however participants will be encouraged to adapt other elements in ways that will work best in their own setting.*

For peer review only - http://bmjopen.bmj.com/site/about/guidelines.xhtml

**describe the changes (what, why, when, and how)**	No major change to the training element of the intervention is anticipated over the course of the trial.
**Item 11. How well (planned): If intervention adherence or fidelity was assessed, describe how and by whom, and if any strategies were used to maintain or improve fidelity, describe them**	Engagement of smokers will be assessed by actors visiting pharmacies and posing as smokers seeing help. Actors will make notes and complete a checklist after the visit.<br>Use of consultation techniques will be assessed by self audio-recording of consultations taking place in the pharmacy consulting room. These recordings will be transcribed and analysed qualitatively.<br>*Engagement and quit rates will be fed back to the advisors by email.*
**Item 12: How well (actual): If intervention adherence or fidelity was assessed, describe the extent to which the intervention was delivered as planned**	Not applicable.

*Features of the initial intervention are in plain text and final intervention in *italics*.

For peer review only
